# Electrochemical synthesis of spirooxindole-pyranopyrazole and spirooxindole-chromene derivatives as inhibitors of acetylcholinesterase

**DOI:** 10.1186/s13065-025-01618-8

**Published:** 2025-09-15

**Authors:** Reem M. Elsapagh, Eman O. Osman, Ahmed M. Hafez, Hala B. El-Nassan

**Affiliations:** 1 Pharmaceutical Organic Chemistry Department, Faculty of Pharmacy, Cairo University, 33 Kasr El-Aini street, Cairo, 11562 Egypt; 2 Department of Biochemistry, School of Life and Medical Sciences, University of Hertfordshire Hosted by Global Academic Foundation, Cairo, 11586 Egypt

**Keywords:** Spirooxindole, Isatin, Pyranopyrazole, Chromene, Electrochemical synthesis, AChE, Acetylcholinesterase inhibitors

## Abstract

An efficient, reliable, and cost-effective approach was applied for the electrochemical synthesis of spirooxindole-pyranopyrazole and spirooxindole-chromene derivatives. The compounds were prepared in high yields and short reaction times by electrochemical synthesis using LiClO_4_ as an electrolyte and Cu/graphite as electrodes. The synthesized products were tested as acetylcholinesterase (AChE) inhibitors. Compounds **4e** and **6b** demonstrated potent inhibitory activity against AChE enzyme with IC_50_ values of 0.51 and 0.84 mM, respectively. Both compounds showed low cytotoxicity and preserved normal cell morphology, confirming their safety. The in-silico study of the ADME properties of compounds **4e** and **6b** revealed a high bioavailability score without affecting any of the CYP isoforms. Kinetic studies were performed to detect the mode of inhibition of the most active compounds, **4e** and **6b.** Also docking studies were performed for both compounds, to evaluate their binding patterns compared to donepezil. The docking and kinetic studies indicated that both compounds inhibited AChE through a competitive mechanism predominantly targeting the catalytic anionic site CAS.

## Introduction

Cholinesterase enzymes, including acetylcholinesterase (AChE) and butyrylcholinesterase (BChE), are essential for the breakdown of acetylcholine, a neurotransmitter that regulates nerve signals [1–4]. AChE is predominantly found at neuromuscular junctions and in the brain, where it quickly hydrolyzes acetylcholine after its release, halting nerve transmission [1]. Cholinesterase inhibitors increase acetylcholine levels, leading to prolonged nerve activity. These inhibitors can be reversible, as those used to treat Alzheimer’s disease and myasthenia gravis, or irreversible, such as those used as pesticides and chemical agents [1, 3, 5]. Therapeutically, cholinesterase inhibitors have various important uses. In Alzheimer’s disease, drugs like donepezil help increase the amount of the neurotransmitter acetylcholine in the brain, which can lead to better memory and cognitive abilities [5–7]. In myasthenia gravis, medications like neostigmine help strengthen muscles by enhancing neuromuscular transmission [8, 9]. Cholinesterase inhibitors are also used to treat conditions like glaucoma, where they aid in reducing the intraocular pressure [10].

**Fig. 1:**
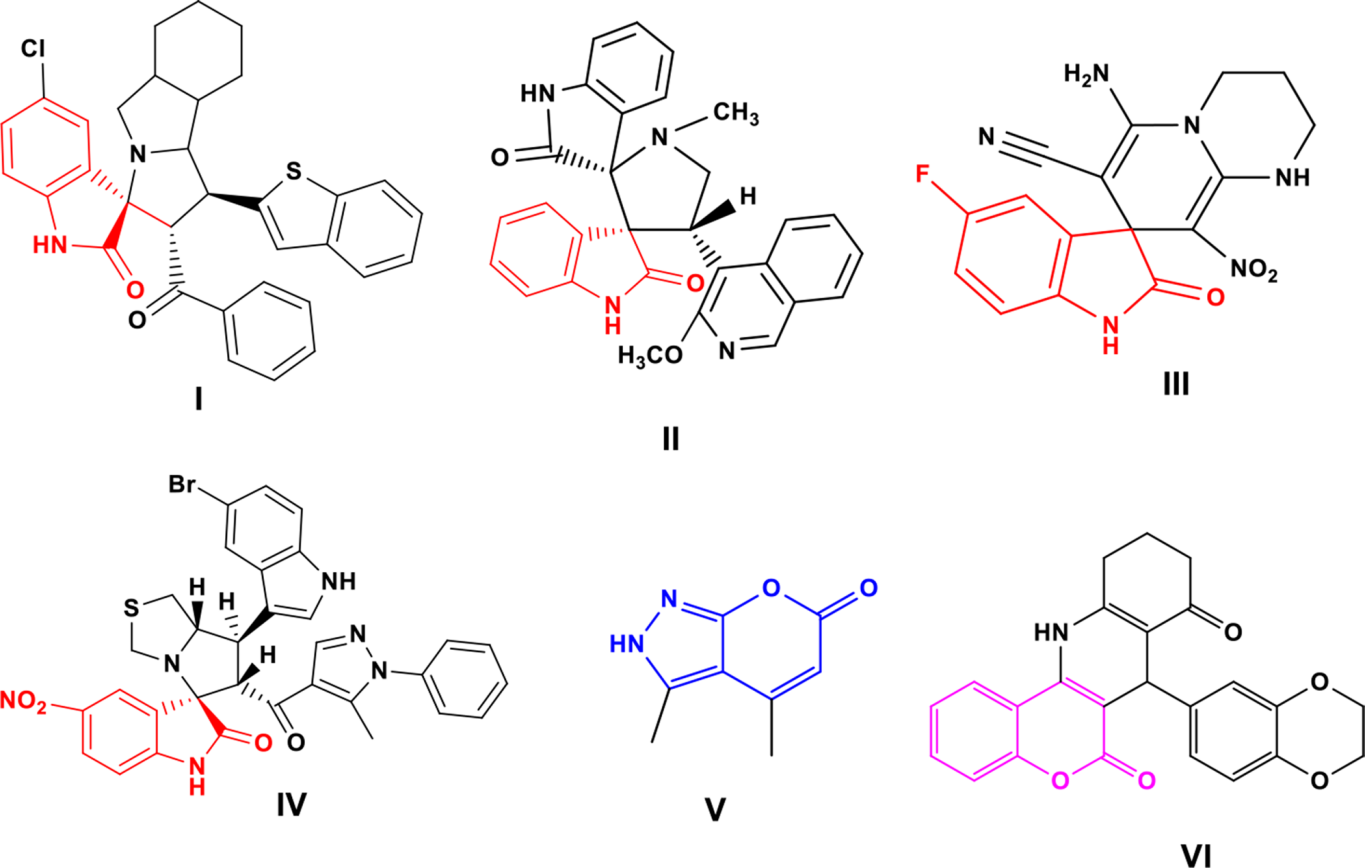
Examples of previously reported AChE inhibitors

Spirooxindoles are known for their activity against AChE [11, 12]. Many researchers focused on synthesizing AChE inhibitors containing spirooxindole hybrids with other active heterocyclic rings. Barakat et al.. provided active spirooxindoles hybrid with octahydroindole and benzothiophene rings **I** (Fig. 1**)**, which showed potential inhibitory activity against AChE (IC_50_ = 20.84 µM/L) [13]. Incorporating a pyrrolidine ring hybridized with spirooxindoles as in compound **II** led to a promising inhibition against AChE enzyme with an IC_50_ of 12.79 µg/L [14]. Maryamabadi et al.. introduced a novel class of spirooxindole-dihydropyridine hybrids **III** that inhibited both AChE and BChE in vitro [15]. Moreover, new spirooxindole-based compounds incorporating an indole ring and a pyrazole ring were developed as potent AChE inhibitors. Among this series, compound **IV** displayed an IC_50_ of 24.1 µM [16]. On the other hand, the pyrano[2,3-*c*]pyrazole derivative **V (**Fig. 1**)** exhibited a promising inhibition against AChE (IC_50_ of 0.38 mg/L) [17]. Furthermore, compound **VI (**Fig. 1**)** with a fused chromene moiety emerged as a potent AChE inhibitor with IC_50_ values of 5.63 µM [18].

The above reports inspired the synthesis of hybrids of spirooxindole core structure with other rings like pyranopyrazole or chromene, aiming to improve their activity as AChE inhibitors.

## Results and discussion

### Design of spirooxindole derivatives as AChE inhibitors

**Fig. 2:**
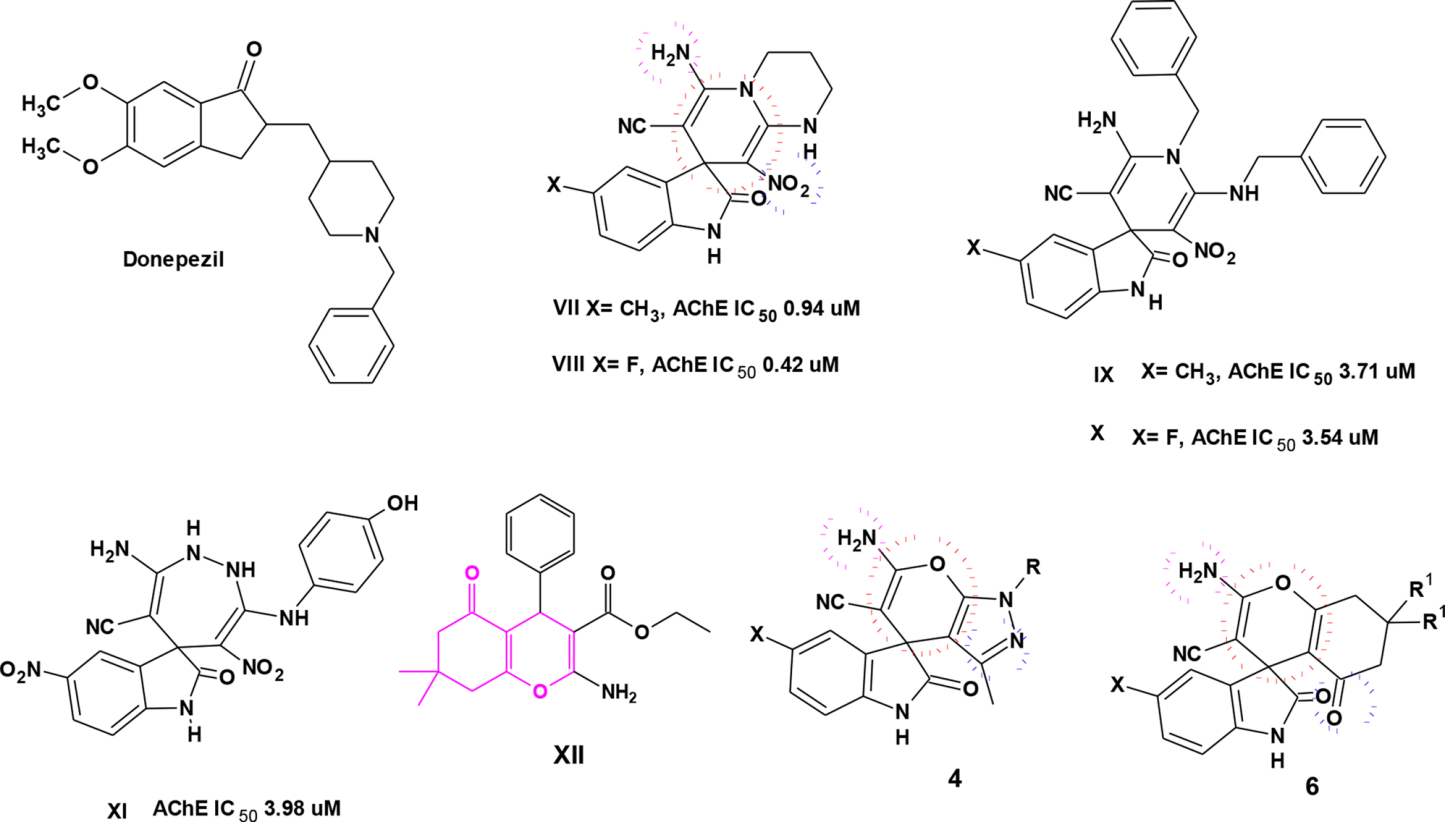
Design of spirooxindole derivatives **4** and **6** as AChE inhibitors

Examining the active site of AChE indicated that it is formed of a peripheral anionic site (PAS) and a catalytic activity site (CAS). Donepezil (Fig. 2), which is one of the most potent AChE inhibitors to date, consists of an indanone ring that occupies the PAS and benzylpyridinium moiety that fills the CAS. The development of novel AChE inhibitors focused on the bioisosteric replacement of the indanone ring with indole or indoline ring that occupied the PAS [19].

In 2016, Maryamabadi et al.. reported the acetyl and butyrylcholinesterase inhibition of a series of spiro-dihydropyridine **VII-X** (Fig. 2). Among the prepared compounds, derivatives **VII** and **VIII** displayed the highest AChE inhibition. The author noticed that the compounds were more potent and more selective inhibitors of AChE than BChE [15]. Moreover, the spiro indolin-1,2-diazepine derivative **XI** was reported as a potent and selective AChE inhibitor with no significant inhibition against BChE [20]. A novel in silico study of 4*H*-chromene derivatives **X** (Fig. 2) as inhibitors of AChE pointed out that both the amino and ester groups can form H-bonds at the PAS, while the aromatic ring can form hydrophobic interactions with the catalytic active site (CAS) [21].

The design of the target compounds in this study focused on the presence of oxindole as it occupied the PAS of AChE. The spiro-oxindole with pyranopyrazole or tetrahydrochromene rings were designed to mimic the structure of compounds **VII** and **VIII**, where pyran ring in compounds **4** and **6** mimicked the size and binding of the pyridine ring in compound **VII** and the nitro group was replaced by either amidic C = N in pyranopyrazole or a carbonyl group in tetrahydrochromene. Both rings were substituted with an amino group that can interact with the CAS site, as in compounds **VII** and **VIII** (Fig. 2).

### Synthesis of spirooxindole derivatives under electrochemical conditions

**Scheme 1:**
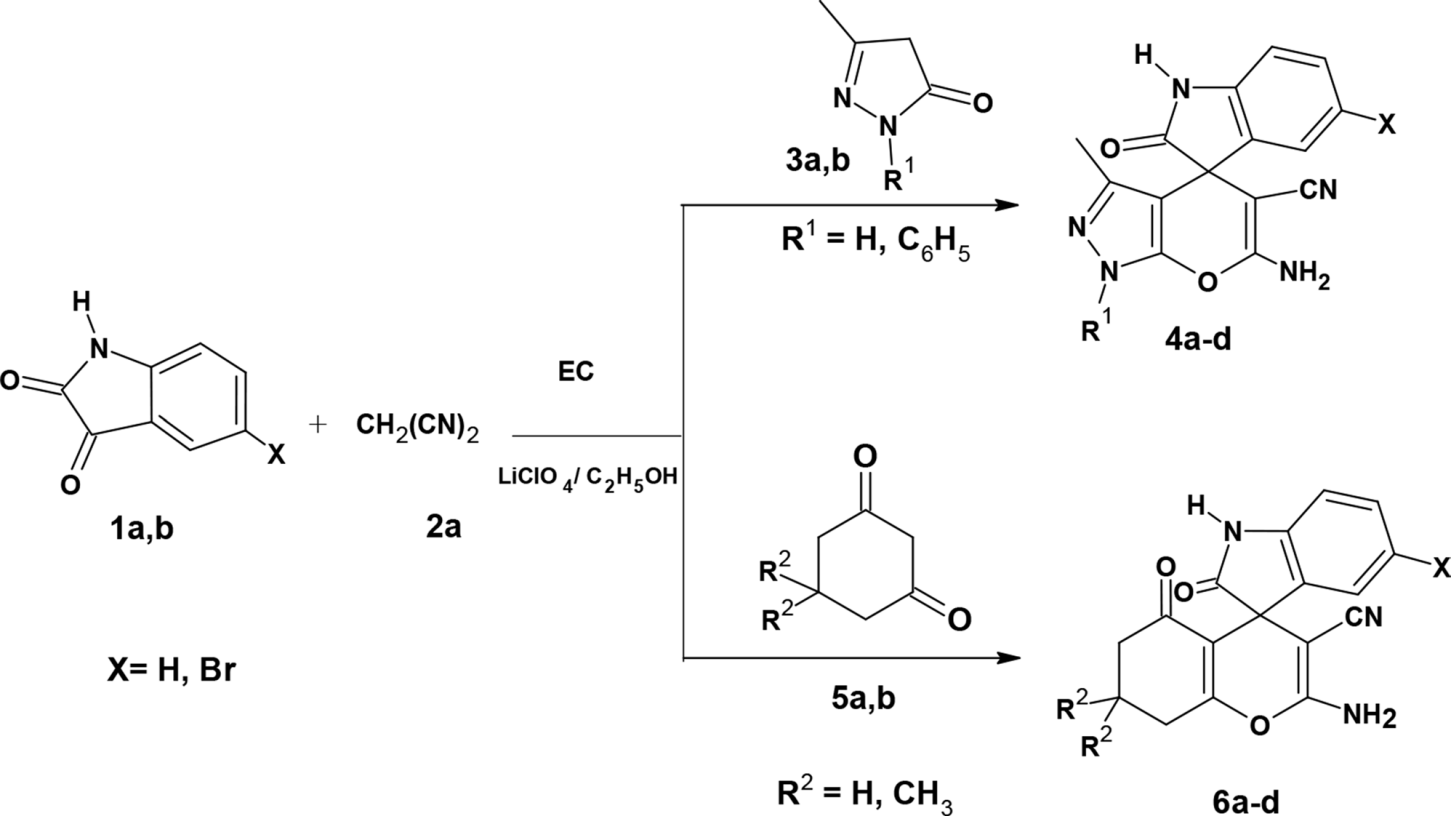
Synthesis of compounds 4a-d and 6a-d

**Scheme 2:**
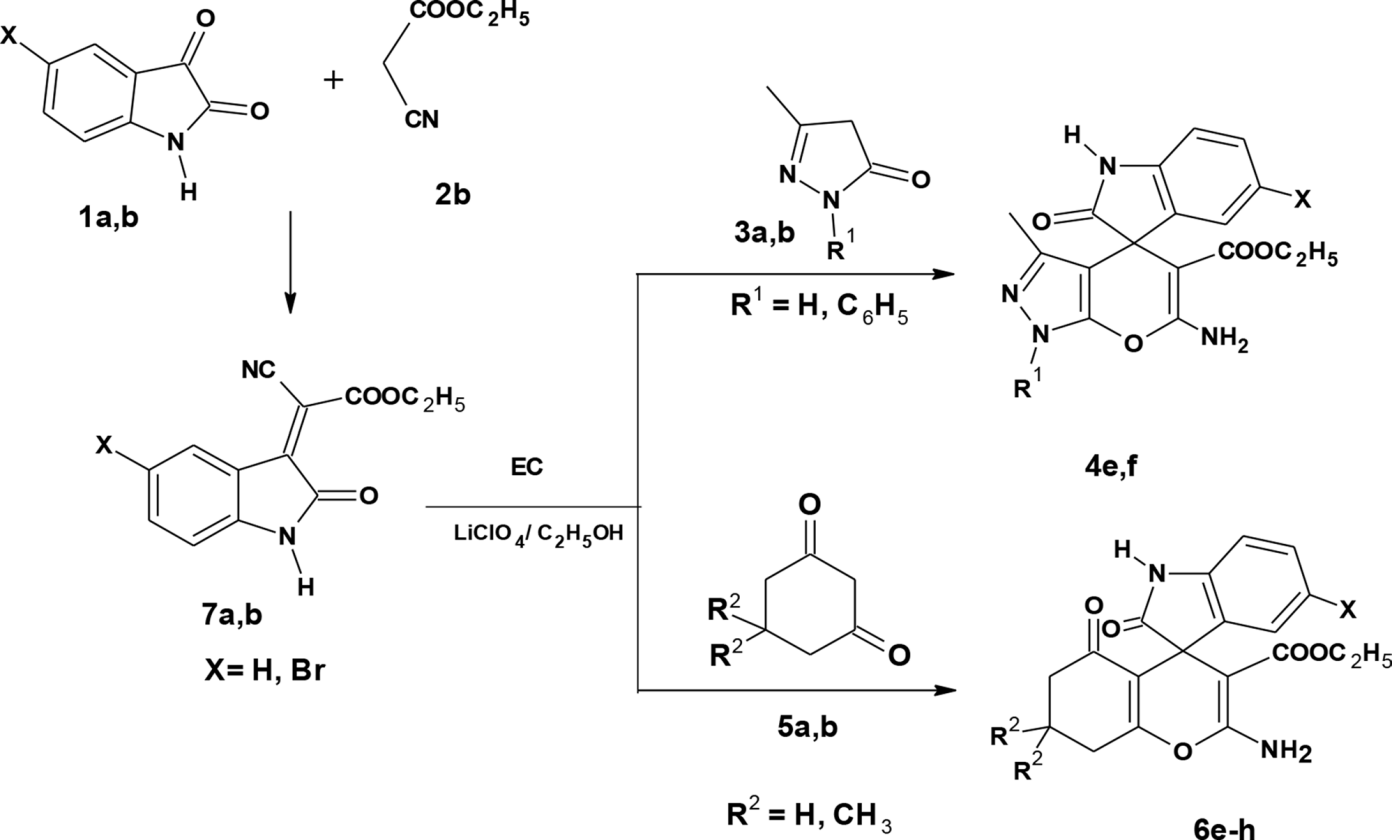
Synthesis of compounds 4e, f and 6e-h

The synthesis pathways were carried out using an electrochemically multicomponent reaction as outlined in Schemes 1 and 2. Electrochemical synthesis offers novel, diverse, green, and environmentally friendly approaches for producing organic compounds on a large scale, owing to its catalytic nature and the use of readily accessible, cost-effective, and environmentally benign reagents [22–26]. Our research group efficiently applied electrochemical synthesis for the synthesis of heterocyclic compounds in water, ethanol, or deep eutectic solvents [27–31].

Searching the literature indicated that spirooxindole-chromene derivatives were synthesized electrochemically through a green one-pot, three-component condensation of cyclic-1,3-diones, malononitrile, and isatins. This sustainable approach utilized an undivided cell with potassium bromide KBr as the electrolyte, an Fe-Mn-O composite cathode, and a graphite anode. The temperature was adjusted to 45 °C, and the reaction was completed in 60 min to afford 83–91% yields of the spirooxindole products [32]. Alternatively, the same reactants were reacted in an undivided cell with sodium bromide as an electrolyte, a graphite or magnesium anode, and an iron cathode at 20 °C for 32 min [33] or at 50 °C for 45–90 min [34] to afford spirooxindole-chromene with yields of 83–98% and 85–98%, respectively. In another study, cyclic-1,3-diketones, malononitrile or ethyl cyanoacetate, and isatins were reacted in an undivided cell using KBr as the electrolyte, iron as a cathode, and magnesium as an anode. The reaction was conducted at 40 °C for 50 min to yield 76–92% of the spirooxindole products [35].

On the other hand, the electrochemical synthesis of spiro[indole-3,4’-pyrano[2,3-*c*]pyrazole] derivatives was performed *via* multicomponent reaction of 3-methyl-2-pyrazolin-5-ones, malononitrile and isatins in an undivided cell using sodium bromide as an electrolyte, iron as a cathode and graphite as an anode at 20^◦^C for 64 min to afford 78–99% yield of the spirooxindole derivatives [36].

In the present work, the electrochemical reaction conditions of the spirooxindole synthesis were optimized via different trials, aiming to obtain high yield in short reaction time and focusing on the use of an undivided cell and economic electrolytes and electrodes. The one-pot three-component synthesis of compound 6’-amino-3’-methyl-2-oxo-1’*H*-spiro[indoline-3,4’-pyrano[2,3-c]pyrazole]-5’-carbonitrile (**4a**) served as a model for optimization of the electrochemical conditions. Thus, isatin **1a** was subjected to the reaction with malononitrile **2a** and pyrazolone **3a** [37] under electrochemical conditions using different solvents, reaction times, electrolytes, and electrodes. A summary of the results is presented in Table 1. The reaction was conducted in ethanol at 70 °C using NaBr as an electrolyte to afford compound **4a** in 76% yield after 45 min (**entry 1**, Table 1). Changing the solvent to acetonitrile decreased the yield to 58% (**entry 2**, Table 1). Similarly, increasing the reaction time to 60 min reduced the yield to 60% (**entry 3**, Table 1). Thus, the optimized reaction conditions were carrying out the electrochemical reaction in ethanol at 70 °C for 45 min. Then, the effect of electrolytes was studied using different electrolytes (**entries 1**,** 4–6**, Table 1). The best result was obtained using lithium perchlorate (LiClO_4_), which afforded compound **4a** in 82% yield after 30 min (**entry 6**, Table 1). Notably, using Cu as a cathode resulted in an 87% yield at 30 min and 93% at 45 min (**entries 7**,**8**, Table 1).

Accordingly, the optimum conditions were conducting the reaction in ethanol at 70 °C for 45 min using LiClO_4_ as an electrolyte and Cu/graphite as electrodes. Using these optimal conditions, compounds **4b**-**4d** were prepared using isatin **1a** or 4-bromoisatin **1b** and 3-methylpyrazolone **3a** or 3-methyl-1-phenylpyrazolone **3b**. Higher yields were obtained using isatin **1a** and 3-methyl pyrazolone **3a (**Table 2**)**.

Likewise, the same reaction conditions were used to prepare 2-amino-7,7-dimethyl-2’,5-dioxo-5,6,7,8-tetrahydrospiro[chromene-4,3’-indoline]-3-carbonitrile derivatives **6a-d** in high yields (80–95%) using cyclohexanedione **5a** or dimedone **5b**, malononitrile **2a**, and isatins derivatives **1a**,** b** as presented in Table 2.

Trials to prepare compounds **4e**-**f** and **6e**-**h** using ethyl cyanoacetate under the same reaction conditions were unsuccessful. Therefore, an alternative method was followed using a two-step procedure. The first step involved reacting isatins **1a**,** b** and ethyl cyanoacetate **2b** in piperidine under reflux to give compounds **7a**,** b** [38]. The second step involved reacting compounds **7a**,** b**, and pyrazolone **3a**,** b** or 1,3-dicarbonyl compounds **5a**,** b** under electrochemical conditions to afford compounds **4e**-**f** and **6e**-**h**, respectively (Scheme 2, Table 3). Notably, it was observed that the reaction of dimedone with malononitrile and isatin gave an excellent yield in a short time. The bromoisatin **1b** gave a lower yield than isatin **1a**, probably due to the moderate solubility of bromoisatin in ethanol.

The syntheses of the spiro[indoline-3,4′-pyrano[2,3-*c*]pyrazole] derivatives **4a**-**4f** were confirmed using IR and NMR spectroscopy. In their FT-IR spectra, the NH_2_ bands were observed at 3363 –3132 cm^− 1^ and the CN band was observed at 2202 –2183 cm^− 1^ in compounds **4a**-**4d**. While in their ^1^H NMR spectra, the NH_2_ protons appeared as a broad singlet signal in the region of δ 7.22–8.22 ppm. While their ^13^C NMR spectra revealed the presence of a C = O signal at δ 177.6-180.1 ppm.

The syntheses of the 2-amino-7,7-dimethyl-2’,5-dioxo-5,6,7,8-tetrahydrospiro[chromene-4,3’-indoline]-3-carbonitrile derivatives **6a-6 h** were also confirmed using IR and ^1^H NMR spectroscopy. In their FT-IR spectra, the NH_2_ bands were observed at 3390 –3143 cm^− 1^. While in their ^1^H NMR spectra, the NH_2_ protons appeared as a broad singlet signal in the region of δ 7.21–7.96. Finally, their ^13^C NMR spectra revealed the presence of a C = O signal at δ 195.1–196 ppm. A detailed description of the spectral data of the prepared compounds is provided in the experimental section.

**Figure:**
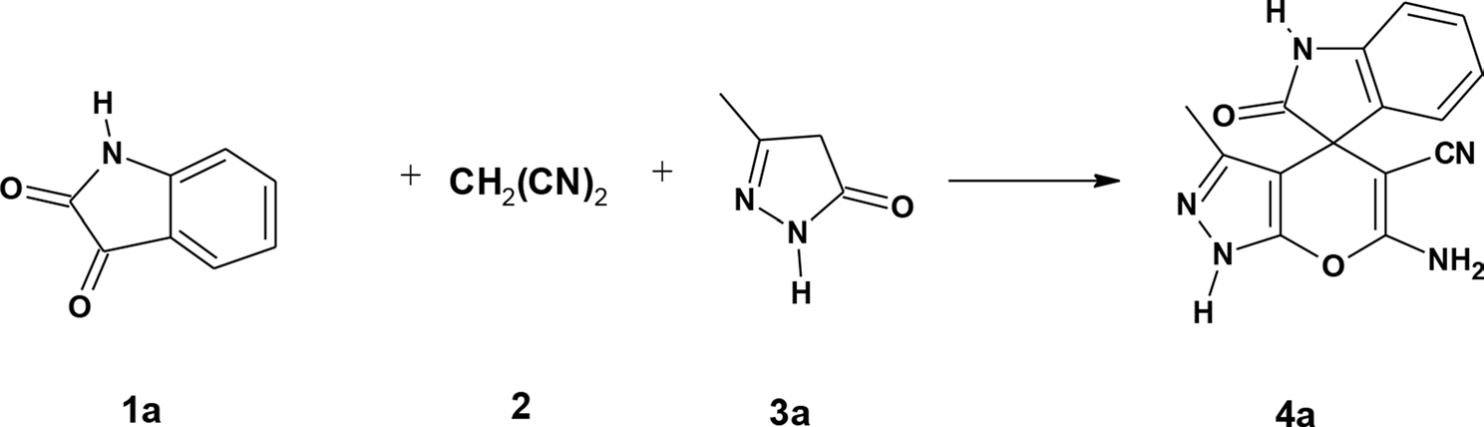

**Table 1:** Synthesis of compound **4a** under electrochemical conditions*

Entry	Solvent	Electrolyte	Time (min)	Electrode Cathode/anode	Yield
1	Ethanol	NaBr	45	graphite/graphite	76%
2	Acetonitrile	NaBr	45	graphite/graphite	58%
3	Ethanol	NaBr	60	graphite/graphite	60%
4	Ethanol	Bu_4_NBF_4_	45	graphite/graphite	65%
5	Ethanol	Bu_4_NClO_4_	45	graphite/graphite	43%
6	Ethanol	LiClO_4_	30	graphite/graphite	82%
7	Ethanol	LiClO_4_	30	copper/graphite	87%
8	Ethanol	LiClO_4_	45	copper/graphite	93%

*Conditions: constant current 30 mA

**Figure:**
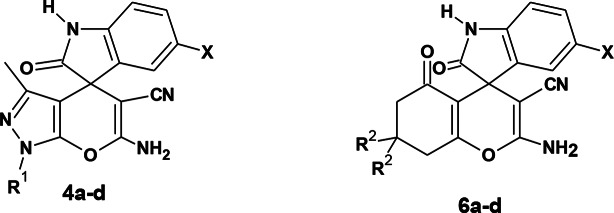

**Table 2:** Electrochemical synthesis of spiro[indoline-3,4′-pyrano[2,3-c] pyrazole] derivatives **4a-d** and 2-amino-2’,5-dioxo-5,6,7,8-tetrahydrospiro[chromene-4,3’-indoline]-3-carbonitrile derivatives 6a-d*

Entry	X	*R*^1^ or *R*^2^	Time (min)	Yield	mp (^0^C)	References
4a	H	H	45	93%	278–280	[36]
4b	Br	H	80	73%	278–280	[39]
4c	H	C_6_H_5_	45	90%	228–230	[36]
4d	Br	C_6_H_5_	60	70%	225–227	[40]
6a	H	H	45	90%	278–280	[32]
6b	Br	H	55	85%	277–279	[33]
6c	H	CH_3_	26	95%	288–290	[32]
6d	Br	CH_3_	35	80%	301–303	[33]

*Conditions: ethanol at 70 °C for 45 min using LiClO_4_ as an electrolyte, Cu/graphite as electrodes and constant current 30 mA

**Figure:**
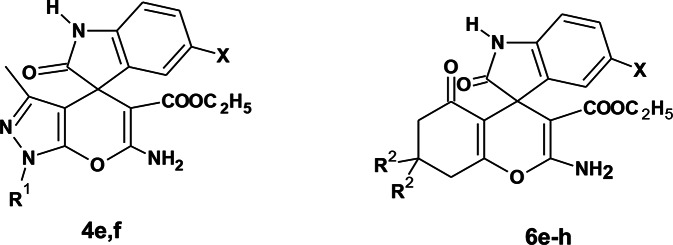

**Table 3:** Electrochemical synthesis of spiro[indoline-3,4′-pyrano[2,3-c] pyrazole] derivatives **4e**, **4f** and 2-amino-2’,5-dioxo-5,6,7,8-tetrahydrospiro[chromene-4,3’-indoline]-3-carbonitrile derivatives **6e-6 h**

Entry	X	*R*^1^ or *R*^2^	Time (min)	Yield	mp (^0^C)	References
4e	H	H	45	75%	281–283	[39]
4f	H	C_6_H_5_	45	80%	236–238	[41]
6e	H	CH_3_	45	86%	256–258	[42]
6f	H	H	45	80%	240–242	[42]
6 g	Br	CH_3_	45	77%	274–276	[40]
6 h	Br	H	45	75%	261–263	[40]

*Conditions: ethanol at 70 °C for 45 min using LiClO4 as an electrolyte, Cu/graphite as electrodes and constant current 30 mA

A comparison between the previously reported electrosynthesis of spirooxindole and the present method is provided in Table 4. The results indicated the benefits of the current procedure in terms of reduced reaction time and the use of readily available, non-sacrificial, and more economic electrodes.

**Table 4:** A comparison between the various reported methods for the electrochemical synthesis of Spirooxindole derivatives and the present study

Method	Temp (^o^C)	Time (min)	Electrolyte	Electrode Cathode/anode	Solvent	Yield	References
1	20	64	NaBr	Iron/graphite	Ethanol	78–99%	[36]
2	50	90	NaBr	Iron/graphite	Propanol	85–98%	[34]
3	45	60	KBr	Fe-Mn-O composite/graphite	Ethanol	83–91%	[32]
4	20	32	NaBr	Iron /magnesium	Ethanol	83–98%	[33]
5	40	50	KBr	Iron /magnesium	Ethanol	76–92%	[35]
6	70	26–80	LiClO_4_	copper/graphite	Ethanol	70–95%	This work

**Fig. 3:**
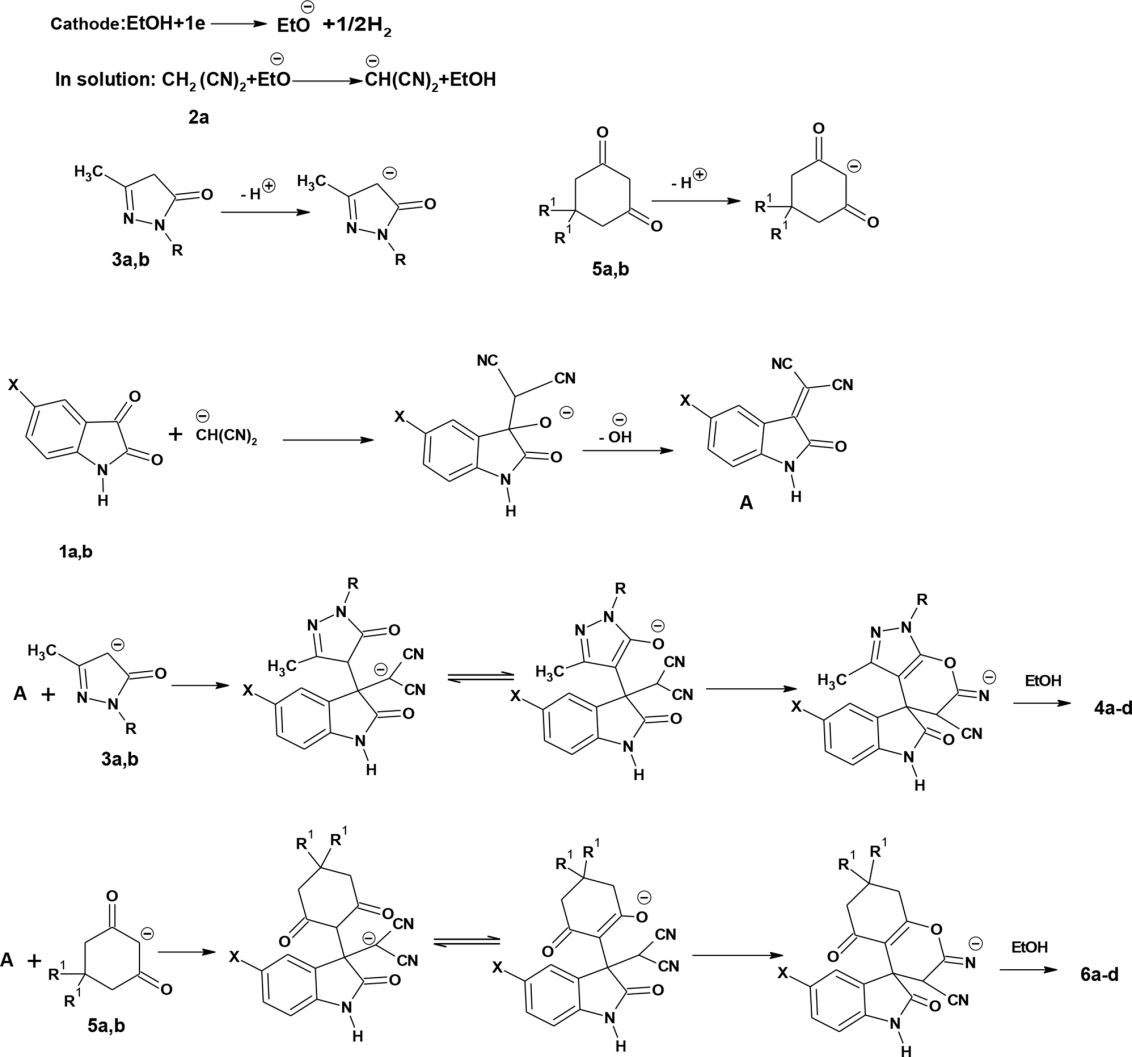
The plausible mechanism of the electrocatalytic synthesis of spirooxindole derivatives

The mechanism of electrochemical synthesis of spirooxindole derivatives is illustrated in Fig. 3. The mechanism involved an initial cathodic reduction of ethanol to form an alkoxide anion, which reacted with malononitrile **2a** to afford the malononitrile anion. The cathodic reduction of 3-methyl-2-pyrazolin-5-ones **3a**,** b** or cyclic 1,3-diketone **5a**,** b** resulted in the formation of their corresponding anions, as reported in our previous work [27].

The Knoevenagel condensation reaction of malononitrile anion and isatins **1a**,** b**, afforded the isatylidenemalononitrile intermediate (**A**). Compound **A** can either react with the anion of 3-methyl-2-pyrazolin-5-ones **3a**,** b** or the anion of cyclic 1,3-diketone **5a**,** b** via Michael addition followed by intramolecular cyclization to afford the target compounds **4a-d** or **6a-d.**

### Biological activity

#### Acetylcholinesterase inhibitory activity

The ability of the synthesized compounds [**4b-4e**,** 6a**,** 6b**,** 6d**,** 6f**,** 6 h**] to inhibit AChE was evaluated adopting the procedure described by Elmann et al. [43]. and Kia et al. [44]. Donepezil was used as a reference, and the percentage inhibition of the tested compounds at 50 µg/mL and 500 µg/mL, as well as the IC_50_ of the most potent compounds, are presented in Table 5.

Concerning the pyranopyrazole series **4b-4f**, it was observed that the amino derivatives **4b-4d** showed low to moderate inhibition percentages. The bromo derivative **4b** showed moderate inhibitory activity at 500 µg/mL, while bromo derivative **4d** showed moderate inhibitory activity at 50 µg/mL. Compound **4c** showed a low inhibitory percentage at 500 µg/mL. However, shifting to the ester-containing derivative **4e**, which has no bromo and no phenyl substituents, resulted in a promising inhibitory percentage (62.47%) at 500 µg/mL with IC_50_ = 0.51 mM. On the other hand, the chromene series **6a-6 h** followed different inhibitory patterns. Concerning the amino derivatives **6a**, **6b**, and **6d**, the unsubstituted derivative **6a** showed moderate inhibition percentage at 500 µg/mL. On the other hand, the bromo derivative **6b** showed moderate activity at 50 µg/mL, but revealed a promising inhibition percentage (61.07%) at 500 µg/mL with IC_50_ = 0.84 mM. However, shifting to the 7,7-dimethyl substituted derivative **6d** resulted in a lowering of the inhibition percentage, especially at 50 µg/mL. In contrast, the ester-containing derivatives **6f** and **6 h** showed low and moderate inhibition percentages at 50 µg/mL and 500 µg/mL, respectively.

The data in Table 5 indicated some insights into the SAR of the compounds as AChE inhibitors. The presence of the ester group enhanced the inhibitory activity compared to the cyano group in the pyranopyrazole series **4b-4f**. Additionally, the presence of the phenyl ring on the pyrazole moiety reduced the inhibition compared to the unsubstituted derivative. The chromene series **6a-6 h** showed better AChE inhibition than the pyranopyrazole series, and better activity was obtained upon using cyclohexandione than upon using dimedone. The presence of a bromo substituent on isatin has little effect on the inhibitory activity.

From the previous results, it can be concluded that these results were comparable to those of spiro-dihydropyridine derivatives **VII** and **VIII** (Fig. 2) [15]. Thus, spirooxindole-pyranopyrazole and spirooxindole-chromene ring systems can act as a lead scaffold for designing more potent AChE inhibitors.

**Table 5:** The results of acetylcholinesterase Inhibition of the Spirooxindole derivatives

Compound	Percentage inhibition at 50 µg/mL	Percentage inhibition at 500 µg/mL	IC_50_ mM
4b	12.49 ± 1.18%	32.70 ± 1.98%	NA*
4c	N/A	7.63 ± 0.58%	NA
4d	22.33 ± 2.25%	N/A	NA
4e	12.17 ± 0.72%	62.47 ± 2.76%	0.51
6a	N/A	32.19 ± 2.91%	NA
6b	43.85 ± 1.78%	61.07 ± 5.36%	0.84
6d	8.39 ± 0.59%	23.29 ± 0.50%	NA
6f	12.77 ± 1.28%	43.70 ± 2.77%	NA
6 h	15.48 ± 0.50%	42.55 ± 3.18%	NA
Donepezil HCl (0.5 µg/mL)	50.23 ± 0.78%		

*NA: not assessed

### Determination of the binding mode to acetylcholinesterase

**Fig. 4:**
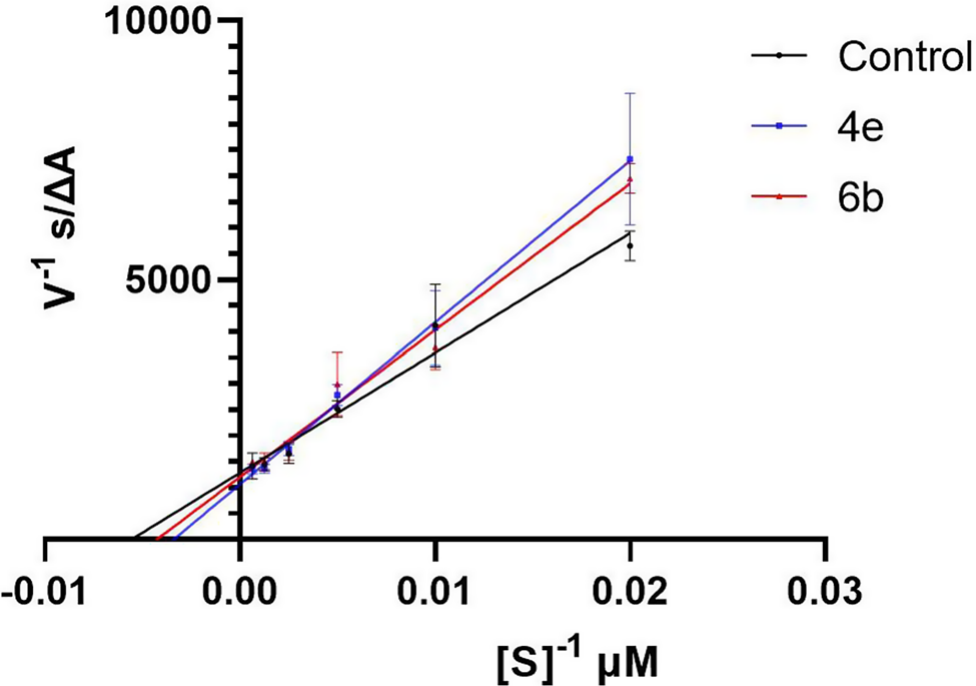
Lineweaver-Burke double reciprocal plot for compounds **4e** and **6b**

To study further the mode of inhibition of compounds **4e** and **6b**, kinetic studies on the electric eel acetylcholinesterase enzyme were performed utilizing Ellman’s method, as mentioned in the experimental section. A Lineweaver-Burk double reciprocal plot was constructed (Fig. 4). The plot showed that the slopes are increasing and that the inhibitors nearly intersect at the y-axis, suggesting a competitive type of enzyme inhibition. Using nonlinear regression and curve fitting in Prism, the software confirmed that the most likely type of inhibition is competitive inhibition (R^2^ = 0.96 for **4e** and 0.93 for **6b**). Provided the 60% sequence identity of the electric eel acetylcholinesterase to the human acetylcholinesterase [45], both compounds will fit in the active site of the enzyme.

### Cytotoxicity study

**Fig. 5:**
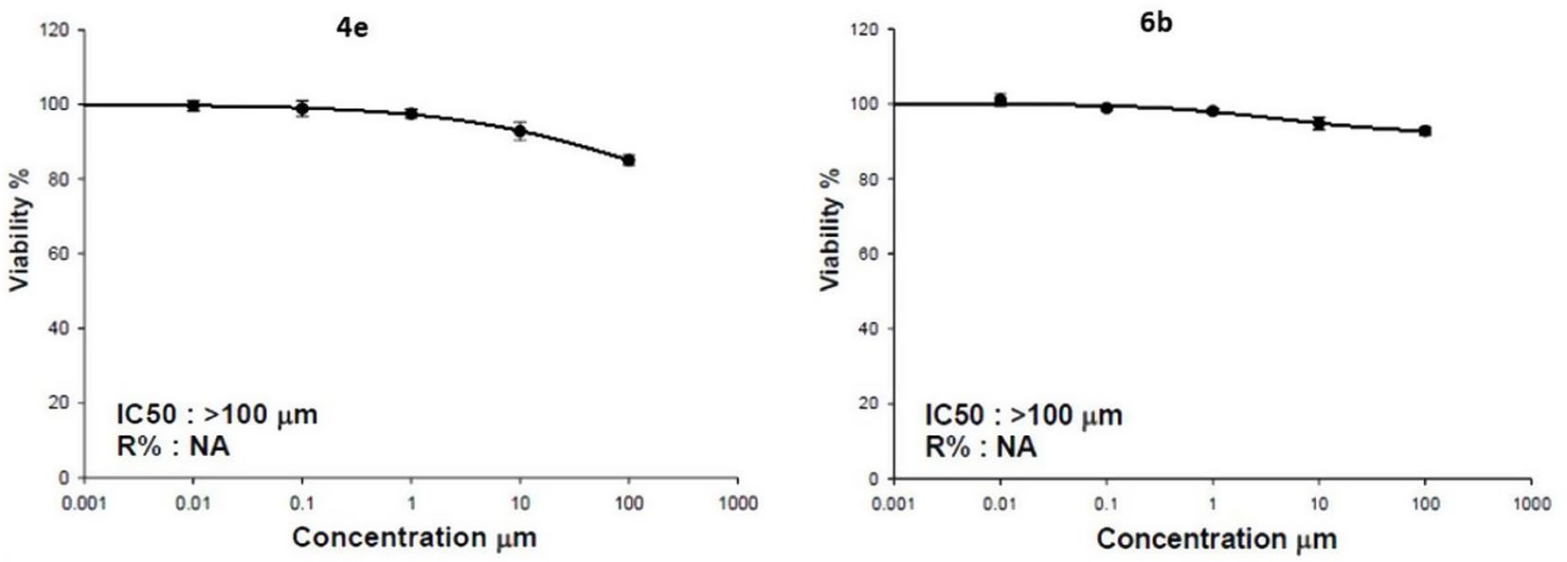
Dose response curve of compounds **4e** and **6b** on Green monkey kidney cell line

Cell viability assays were performed to evaluate the potential cytotoxicity of compounds **4e** and **6b** on green monkey kidney (vero) cell line. The results demonstrated that both compounds exhibited no significant cytotoxic effects, as their IC₅₀ values were greater than 100 µM, indicating a favorable safety profile. At the highest tested concentration (100 µM), the percentage of viable cells remained high, with **4e** and **6b** showing viability rates of 85.01 ± 1.34% and 92.75 ± 1.01%, respectively (Fig. 5).

**Fig. 6:**
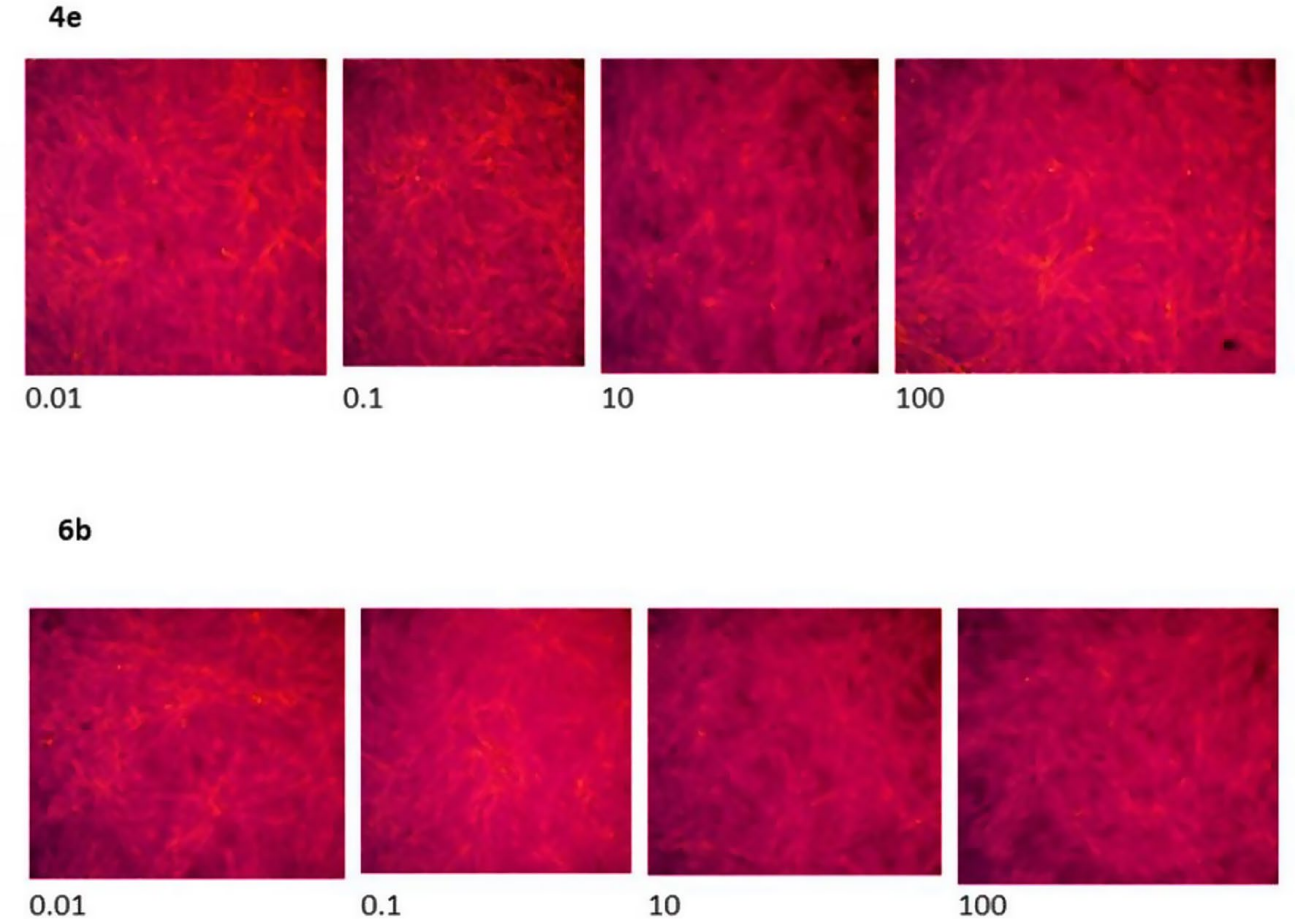
Microscopic examination of cells when treated with compounds **4e** and **6b** at various concentrations (0.01–100 µM)

In addition to quantitative MTT assay results, microscopic examination of green monkey kidney cell morphology was conducted following treatment with compounds **4e** and **6b** at various concentrations (0.01–100 µM). As shown in Fig. 6, the treated cells retained their normal morphology, with no evidence of cell abnormalities. Even at the highest concentration tested (100 µM), no significant morphological abnormalities were observed in cells treated with either compound. These observations further confirm the safety of compounds **4e** and **6b.**

### In *silico* prediction of physicochemical, ADME, and pharmacokinetic properties

The SwissADME web tool (http://www.swissadme.ch), provided by the Swiss Institute of Bioinformatics (SIB), was utilized to analyze the physicochemical properties, ADME profiles, pharmacokinetic characteristics, and drug-like nature of the most potent synthesized derivatives, namely **4e** and **6b**.

The prediction results are summarized in Table 6. Interestingly, both compounds **4e** and **6b** demonstrated high expected GIT absorption despite the poor water solubility that was predicted from their log O/W values. Compound **4e** showed no inhibitory effect on the panel of the five CYP isoforms: CYP1A2, CYP2C19, CYP2C9, CYP2D6, and CYP3A4. Compound **6b** inhibited three CYP isoforms: CYP2C9, CYP2C19, and CYP3A4. Furthermore, compounds **4e** and **6b** satisfied the drug-like criteria defined by the major drug companies: Lipinski (Pfizer), Ghose, Veber (GSK), Egan (Pharmacia), or Muegge (Bayer). Furthermore, a high bioavailability score (0.56) was observed for the target compounds **4e** and **6b**.

**Fig. 7:**
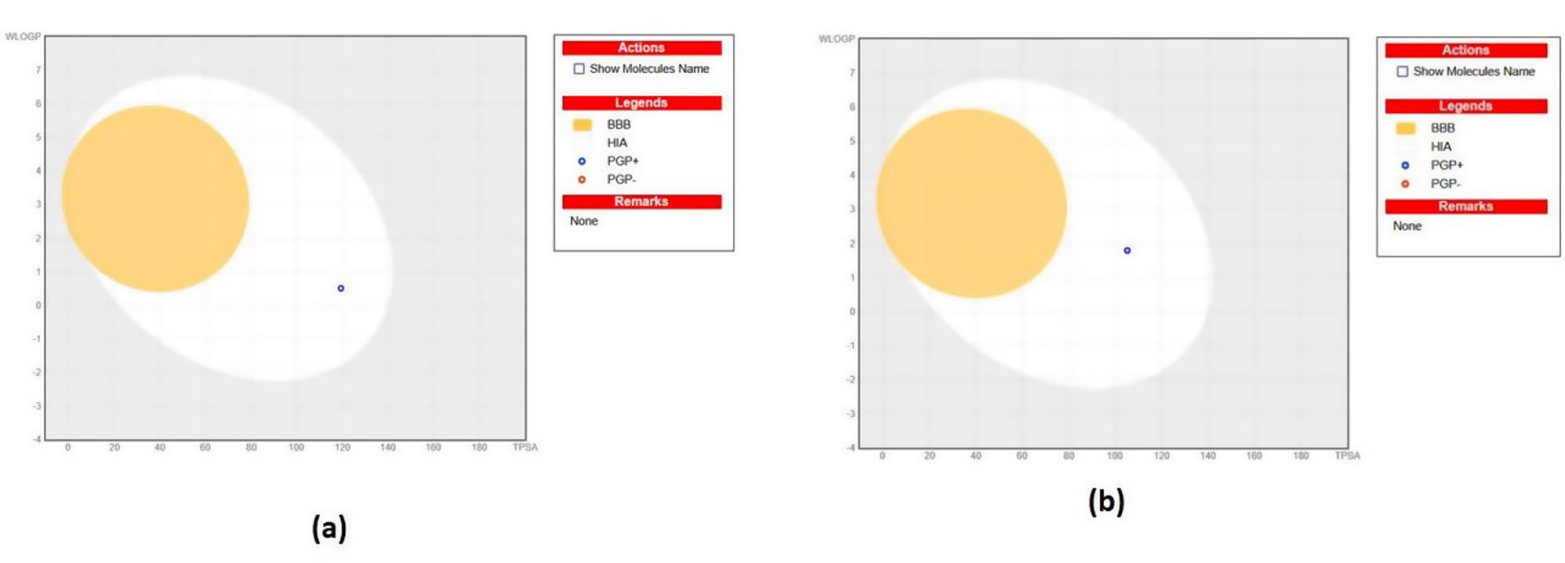
Boiled egg representation of (**a**) compound **4e** and (**b**) compound **6b**

A BOILED-Egg plot, which displays the WLOGP against the topological polar surface area for the tested compounds, is presented in (Fig. 7). Because the BBB was not permeable and compounds **4e** and **6b** lay in the human intestinal absorption zone, the likelihood of anticipated CNS adverse effects was decreased. Thus, it can be concluded that these substances might have potentially appealing pharmacokinetic and physicochemical characteristics in addition to their promising biological action.

**Table 6:** Molecular characteristics of compounds **4e** and **6b** using the Swiss ADME website (http://www.swissadme.ch)

Parameters	4e	6b
Consensus Log P	1.24	1.72
Water solubility (ESOL)	Poorly soluble	Poorly soluble
GI absorption	High	High
BBB permeant	No	No
P-gp substrate	Yes	yes
CYP1A2 inhibitor	No	No
CYP2C19 inhibitor	No	Yes
CYP2C9 inhibitor	No	Yes
CYP2D6 inhibitor	No	No
CYP3A4 inhibitor	No	Yes
Lipinski	Yes	Yes
Ghose	Yes	Yes
Veber	Yes	Yes
Egan	Yes	Yes
Muegge	Yes	Yes
Bioavailability Score	0.56	0.56

### Molecular docking study

The active center of AChE lies at the base of a ~ 20 Å aromatic gorge and comprises two functional subsites. The catalytic anionic site (CAS) houses the Ser203–His447–Glu334 triad and the key cationπ anchor Trp86. The peripheral anionic site (PAS); which is defined principally by Tyr72, Tyr124, Tyr337, and Trp286, guides substrates into the gorge and is implicated in amyloidβ binding [46, 47].

**Fig. 8:**
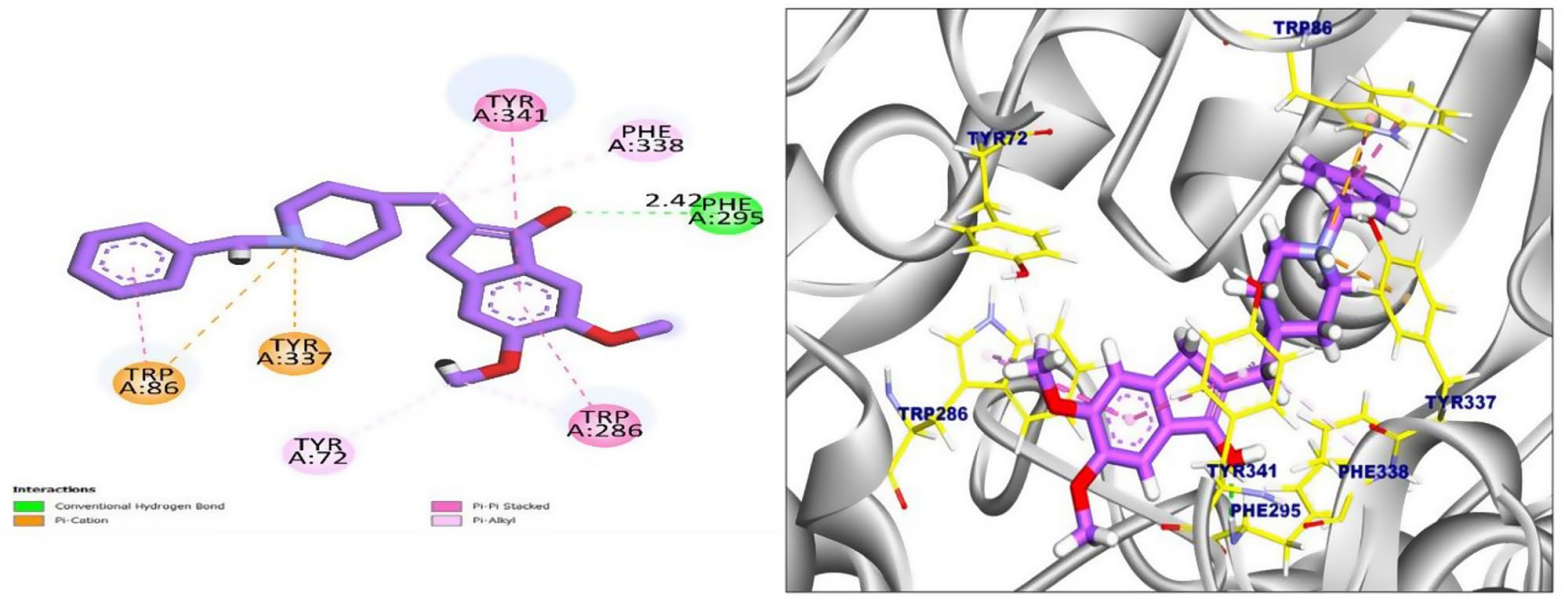
The 2D interactions of the redocked co-crystallized donepezil in the active site of human acetylcholinesterase (left) and the 3D interactions of the redocked co-crystallized donepezil in the active site (right)

In this work, docking study of the most active compounds **4e** and **6b** was performed using Autodock vina to evaluate their binding patterns compared to donepezil. The docking of donepezil, a known dual-binding site AChE inhibitor, revealed a binding energy of − 8.28 kcal/mol. It formed one hydrogen bond with Phe295 at 2.41 Å and was stabilized by multiple hydrophobic interactions, including π–π and π–cation stacking with residues such as Trp86, Tyr72, Tyr337, Tyr341, Phe338, and Trp286. These interactions spanned both the CAS and PAS, supporting the role of donepezil as a dual-site inhibitor with high affinity and potential anti-amyloid activity (Fig. 8).

**Fig. 9:**
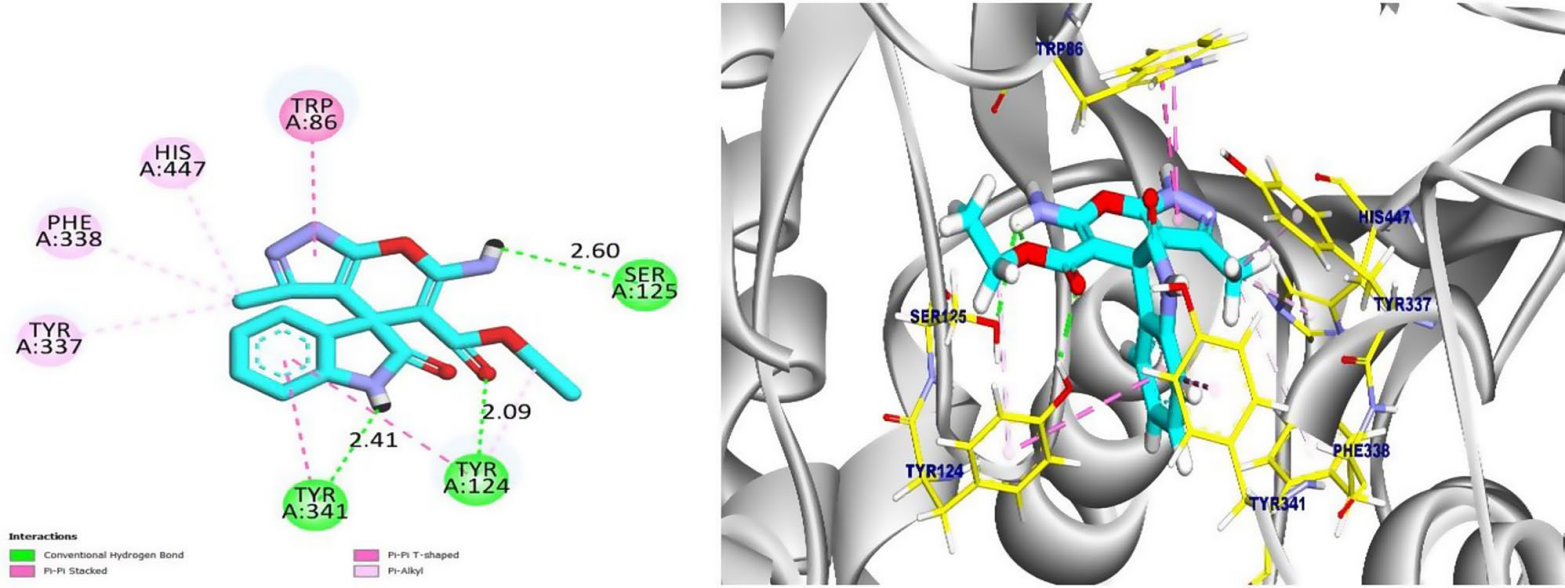
The 2D representation of **4e** interactions with the active site of human acetylcholinesterase (left) and the 3D representation of **4e** interactions in the active site (right)

**Fig. 10:**
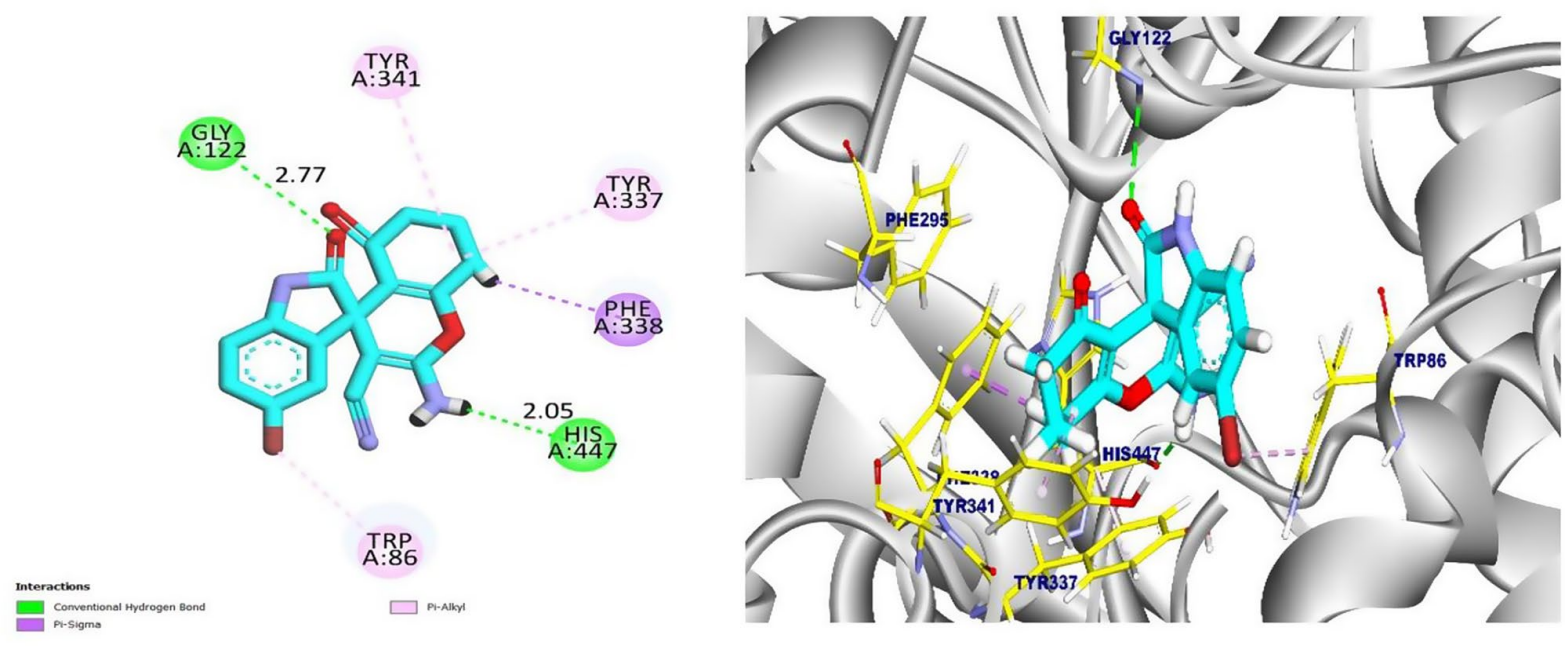
The 2D representation of **6b** interactions with the active site human acetylcholinesterase (left) and the 3D representation of **6b** interactions in the active site (right)

Compound **4e** showed the most favorable binding affinity of − 8.63 kcal/mol. It established seven strong hydrophobic interactions with key residues in the active site, with Trp86, His447, Phe338, Tyr337, and Tyr341. Additionally, compound **4e** formed three hydrogen bonds with Ser125 (2.60 Å), Tyr124 (2.09 Å), and Tyr341 (2.41 Å), which contributed to its high binding stability. Compound **4e** appears to fit deeply into the catalytic site, interacting tightly with CAS and PAS residues (Fig. 9). In contrast, compound **6b** exhibited a slightly lower binding affinity of − 7.25 kcal/mol. It formed two hydrogen bonds with Gly122 and His447 at 2.77 Å and 2.05 Å, respectively, along with four hydrophobic interactions with Phe338, Tyr337, Tyr341, and Trp86. The results revealed that compound **6b** remains largely confined to the CAS and forms fewer peripheral contacts, which accounts for its lower overall affinity (Fig. 10). These docking results validate the original design by showing that the spiro-oxindole scaffold, particularly in compound **4e**, efficiently binds AChE’s CAS and PAS, resulting in a high binding affinity.

Interestingly, these docking results aligned well with the kinetic study results, where Lineweaver–Burk plots suggested competitive inhibition. The competitive inhibition observed for compound **4e** was strongly supported by its substantial interactions with CAS residues, particularly Trp86 and His447. Compound **4e** also interact with PAS residues such as Tyr124 and Tyr341; these interactions served to enhance binding stability and positioning within the active-site gorge, without changing the competitive nature of the inhibition. Likewise, the inhibition mechanism of compound **6b** aligned well with its docking profile, which exhibited a predominant interaction with CAS residues and minimal involvement of PAS. This supported its role as a classical competitive inhibitor.

### Experimental section

#### General remarks

Melting points were obtained on a Griffin apparatus and were represented as uncorrected values. C, H, and N microanalyses were carried out at the Regional Center for Mycology and Biotechnology, Faculty of Pharmacy, Al-Azhar University. IR spectra were recorded in cm^− 1^ values on Shimadzu IR 435 spectrophotometer (Shimadzu Corp., Kyoto, Japan), Faculty of Pharmacy, Cairo University. ^1^H NMR spectra were performed on Bruker 400 MHz (Bruker Corp., Billerica, MA, USA) spectrophotometer, Faculty of Pharmacy, Cairo University. The chemical shifts were recorded in ppm on *δ* scale, coupling constants (*J*) were given in Hz, and peak multiplicities were designated as follows: s, singlet; d, doublet; dd, doublet of doublets; t, triplet; m, multiplet. ^13^C NMR spectra were carried out on Bruker 100 MHz spectrophotometer, Faculty of Pharmacy, Cairo University. The progress of the reactions was monitored by TLC on silica gel sheets (MERCK 60 F 254) and followed up by UV lamp visualization. The original NMR spectra of the investigated compounds are provided as supporting information.

#### Electrochemical synthesis of spiro[indoline-3,4′-pyrano[2,3-*c*] pyrazole] (4a)

A mixture of isatin **1a** (2 mmol), malononitrile **2a** (2 mmol), and the pyrazolone derivative **3a** [37] (2 mmol) was dissolved in the appropriate solvent (10 mL) in an undivided cell fitted with different electrodes as indicated in Table 1. The reaction proceeded at 70 °C using a constant current (30 mA) for different times as described in Table 1. After completing the reaction as indicated by TLC, the reaction mixture was cooled, and water (20 mL) was added. After precipitation, the solid was collected by filtration and recrystallized from ethanol.

#### Electrochemical synthesis of spiro[indoline-3,4′-pyrano[2,3-*c*] pyrazole] (4b-4d)

A mixture of isatin **1a** or bromoisatin **1b** (2 mmol), malononitrile **2a** (2 mmol) and the pyrazolone derivative **3a** [37] or **3b** [48] (2 mmol) was dissolved in ethanol (10 mL) in an undivided cell fitted with copper as a cathode and graphite as an anode. The reaction was conducted at 70 °C using a constant current (30 mA). After completing the reaction as indicated by TLC, the reaction mixture was cooled, and water (20 mL) was added. The precipitate formed was filtered and recrystallized from ethanol.

#### 6’-Amino-3’-methyl-2-oxo-1’H-spiro[indoline-3,4’-pyrano[2,3-*c*]pyrazole]-5’-carbonitrile (4a)

IR (KBr): 3336 − 3132 (NH_2_, NH), 2183 (CN), 1712 (C = O) cm^− 1^; ^1^H NMR (400 MHz, DMSO-*d*_*6*_): δ 1.54 (s, 3 H, CH_3_), 6.90–6.92 (d, *J =* 7.6 Hz, 1H, ArH), 6.97–7.05 (m, 2 H, ArH), 7.22–7.26 (m, 3 H, ArH + NH_2_, D_2_O exchangeable), 10.59 (s, 1H, NH, D_2_O exchangeable), 12.28 (s, 1H, NH, D_2_O exchangeable) ppm; ^13^C NMR (100 MHz, DMSO-*d*_*6*_): δ 9.4 (CH_3_), 47.7 (C-4**’**), 55.6 (C-5**’**), 95.8, 110.1, 119.2, 123.0, 124.9, 129.3, 133.1, 135.2, 141.9, 155.7, 162.9, 178.5 (C = O) ppm; Anal. Calcd for C_15_H_11_N_5_O_2_ (293.29): C, 61.43; H, 3.78; N, 23.88; Found: C, 61.62; H, 3.94; N, 24.05.

#### 6’-Amino-5-bromo-3’-methyl-2-oxo-1’H-spiro[indoline-3,4’-pyrano[2,3-c] pyrazole]-5’-carbonitrile (4b)

IR (KBr): 3356 − 3344 (NH_2_, NH), 2183 (CN), 1693 (C = O) cm^− 1^; ^1^H NMR (400 MHz, DMSO-*d*_*6*_): δ 1.59 (s, 3 H, CH_3_), 6.88–6.90 (d, *J* = 8.2 Hz, 1H, ArH), 7.24–7.30 (m, 3 H, ArH + NH_2_, D_2_O exchangeable), 7.44–7.45 (m, 1H, ArH), 10.76 (s, 1H, NH, D_2_O exchangeable), 12.34 (s, 1H, NH, D_2_O exchangeable) ppm; ^13^C NMR (100 MHz, DMSO-*d*_*6*_): δ 9.5 (CH_3_), 48.0 (C-4**’**), 55.0 (C-5**’**), 95.2, 112.2, 114.7, 119.1, 127.8, 132.2, 135.3, 135.5, 141.2, 155.6, 163.0, 178.1 (C = O) ppm; Anal. Calcd for C_15_H_10_BrN_5_O_2_ (372.18): C, 48.41; H, 2.71; N, 18.82; Found: C, 48.67; H, 2.96; N, 19.05.

#### 6’-Amino-3’-methyl-2-oxo-1’-phenyl-1’H-spiro[indoline-3,4’-pyrano[2,3-c] pyrazole]-5’-carbonitrile (4c)

IR (KBr): 3294 − 3174 (NH_2_, NH), 2194 (CN), 1701 (C = O) cm^− 1^; ^1^H NMR (400 MHz, DMSO-*d*_*6*_): δ 1.55 (s, 3 H, CH_3_), 6.94–6.96 (d, *J* = 7.7 Hz, 1H, ArH), 7.01–7.05 (t, *J* = 7.2 Hz, 1H, ArH), 7.17–7.19 (d, *J* = 7.2 Hz, 1H, ArH), 7.27–7.37 (m, 2 H, ArH), 7.50–7.56 (m, 4 H, 2ArH + NH_2_, D_2_O exchangeable), 7.78–7.80 (d, *J* = 7.76 Hz, 2 H, ArH), 10.73 (s, 1H, NH, D_2_O exchangeable) ppm; ^13^C NMR (100 MHz, DMSO-*d*_*6*_): δ 12.1 (CH_3_), 48.2 (C-4**’**), 56.6 (C-5**’**), 96.7, 110.3, 118.4, 120.6, 123.1, 125.3, 127.0, 129.7, 129.9, 132.5, 137.6, 142.0, 144.4, 145.3, 161.5, 178.0 (C = O) ppm; Anal. Calcd for C_21_H_15_N_5_O_2_ (369.38): C, 68.28; H, 4.09; N, 18.96; Found: C, 68.41; H, 4.28; N, 19.17.

#### 6’-Amino-5-bromo-3’-methyl-2-oxo-1’-phenyl-1’H-spiro[indoline-3,4’-pyrano[2,3-c] pyrazole]-5’-carbonitrile (4d)

IR (KBr): 3363 − 3317, 3186 (NH_2_, NH), 2202 (CN), 1705 cm^− 1^; ^1^H NMR (400 MHz, DMSO-*d*_*6*_): δ 1.60 (s, 3 H, CH_3_), 6.91–6.93 (d, *J* = 8.7 Hz, 1H, ArH), 7.34–7.38 (t, *J* = 7.4 Hz, 1H, ArH), 7.46–7.54 (m, 4 H, ArH), 7.63 (s, 2 H, NH_2_, D_2_O exchangeable), 7.78–7.80 (d, *J* = 7.8 Hz, 2 H, ArH), 10.89 (s, 1H, NH, D_2_O exchangeable) ppm; ^13^C NMR (100 MHz, DMSO-*d*_*6*_): δ 12.21 (CH_3_), 48.4 (C-4**’**), 56.0 (C-5**’**), 96.1, 112.3, 118.3, 120.7, 127.0, 128.3, 129.8, 132.5, 135.1, 137.6, 141.2, 144.2, 145.5, 161.5, 170.8, 177.6 (C = O) ppm; Anal. Calcd for C_21_H_14_BrN_5_O_2_ (448.28): C, 56.27; H, 3.15; N, 15.62; Found: C, 56.43; H, 3.26; N, 15.89.

#### Synthesis of 2-oxo-(3*H*)-indol-3-ylidene Ethyl cyanoacetate (7a) and 5-bromo-2-oxo-(3*H*)-indol-3-ylidene Ethyl cyanoacetate (7b) [38]

A mixture of isatin **1a** or bromoisatin **1b (**0.006 mmol**)** and ethyl cyanoacetate **2b** (0.006 mmol**)** in piperidine (0.7 mL) was heated under reflux for 5 min. The solid formed was filtered, dried and used without further purification.

#### Electrochemical synthesis of spiro[indoline-3,4′-pyrano[2,3-c] pyrazole] (4e and 4f)

A mixture of compound **7a** [38] (0.002 mmol) and pyrazolone **3a (**0.002 mmol**)** was dissolved in ethanol (10 mL) in an undivided cell fitted with copper as a cathode and graphite as an anode. The reaction was conducted at 70 °C using a constant current 30 mA. After completing the reaction as indicated by TLC, the reaction mixture was cooled, and water (20 mL) was added. The precipitate formed was filtered and recrystallized from ethanol.

#### Ethyl 6’-amino-3’-methyl-2-oxo-1’H-spiro[indoline-3,4’-pyrano[2,3-c]pyrazole]-5’-carboxylate (4e)

IR (KBr): 3352 − 3259, 3157 (NH_2_, NH), 1705, 1670 (C = O) cm^− 1^; ^1^H NMR (400 MHz, DMSO-*d*_*6*_): δ 0.69–0.73 (t, *J =* 7.1 Hz, 3 H, *CH*_*3*_CH_2_), 1.57 (s, 3 H, CH_3_), 3.66–3.74 (m, 2 H, CH_3_*CH*_*2*_), 6.81–6.88 (m, 3 H, ArH), 7.10–7.15 (m, 1H, ArH), 8.01 (s, 2 H, NH_2_, D_2_O exchangeable), 10.36 (s, 1H, NH, D_2_O exchangeable), 12.14 (s, 1H, NH, D_2_O exchangeable) ppm; ^13^C NMR (100 MHz, DMSO-*d*_*6*_): δ 9.3 (CH_3_), 13.5 (*CH*_*3*_CH_2_), 47.5 (C-4**’**), 59.1 (CH_3_*CH*_*2*_), 74.6 (C-5**’**), 97.5, 109.1, 122.1, 123.0, 127.7, 135.1, 137.1, 142.3, 154.8, 163.3, 168.6, 180.1 (C = O) ppm; Anal. Calcd for C_17_H_16_N_4_O_4_ (340.34): C, 60.00; H, 4.74; N, 16.46; Found: C, 60.21; H, 4.89; N, 16.65.

#### Ethyl 6’-amino-3’-methyl-2-oxo-1’-phenyl-1’H-spiro[indoline-3,4’-pyrano[2,3-c]pyrazole]-5’-carboxylate (4f)

IR (KBr): 3352 − 3190 (NH_2_, NH), 1697, 1643 (C = O) cm^− 1^; ^1^H NMR (400 MHz, DMSO-*d*_*6*_): δ 0.73–0.77 (t, *J* = 7.2 Hz, 3 H, *CH*_*3*_CH_2_), 1.60 (s, 3 H, CH_3_), 3.71–3.75 (q, *J* = 7.2 Hz, 2 H, CH_3_*CH*_*2*_), 6.86–6.98 (m, 3 H, ArH), 7.16–7.19 (t, *J* = 7.6 Hz, 1H, ArH), 7.31–7.35 (t, *J* = 7.6 Hz, 1H, ArH), 7.45–7.53 (m, 2 H, ArH), 7.81–7.83 (d, *J* = 8.0 Hz, 2 H, ArH), 8.22 (s, 2 H, NH_2_, D_2_O exchangeable), 10.51 (s, 1H, NH, D_2_O exchangeable) ppm; ^13^C NMR (100 MHz, DMSO-*d*_*6*_): δ 11.9 (CH_3_), 13.6 (*CH*_*3*_CH_2_), 48.0 (C-4**’**), 59.7 (CH_3_*CH*_*2*_), 75.0 (C-5**’**), 98.4, 109.6, 120.2, 122.5, 123.4, 124.7, 127.7, 128.5, 129.9, 136.0, 137.4, 142.2, 144.9, 161.7, 168.4, 180.1 (C = O) ppm; Anal. Calcd for C_23_H_20_N_4_O_4_ (416.44): C, 66.34; H, 4.84; N, 13.45; Found: C, 66.24; H, 5.02; N, 13.72.

#### Electrochemical synthesis of 2-amino-7,7-dimethyl-2’,5-dioxo-5,6,7,8-tetrahydrospiro[chromene-4,3’-indoline]-3-carbonitrile derivatives (6a-6d)

A mixture of isatin **1a** or bromoisatin **1b** (2 mmol), malononitrile **2a** (2 mmol) and cyclohexanedione **3a** or dimedone **3b** (2 mmol) was dissolved in ethanol (10 mL) in an undivided cell fitted with copper as a cathode and graphite as an anode. The reaction was conducted at 70 °C using a constant current 30 mA. After completing the reaction indicated by TLC, the reaction mixture was cooled, and water (20 mL) was added. The precipitate formed was filtered and recrystallized from ethanol.

#### 2-Amino-2’,5-dioxo-5,6,7,8-tetrahydrospiro[chromene-4,3’-indoline]-3-carbonitrile (6a)

IR (KBr): 3367 − 3298, 3159 (NH_2_, NH), 2194 (CN), 1716, 1658 (C = O) cm^− 1^; ^1^H NMR (400 MHz, DMSO-*d*_*6*_): δ 1.90–1.94 (t, *J* = 6.0 Hz, 2 H, CH_2_), 2.18–2.27 (m, 2 H, CH_2_), 2.64–2.67 (t, *J* = 6.0 Hz, 2 H, CH_2_), 6.77–6.79 (d, *J* = 7.6 Hz, 1H, ArH), 6.87–6.91 (t, *J* = 7.3 Hz, 1H, ArH), 7.00-7.01 (d, *J* = 7.2 Hz, 1H, ArH), 7.12–7.16 (t, *J* = 7.6 Hz, 1H, ArH), 7.21 (s, 2 H, NH_2_, D_2_O exchangeable), 10.39 (s, 1H, NH, D_2_O exchangeable) ppm; ^13^C NMR (100 MHz, DMSO-*d*_*6*_): δ 20.2 (C-7), 27.2 (C-8), 36.8 (C-6), 47.3 (C-4), 58.0 (C-3), 109.6, 112.3, 117.8, 122.1, 123.6, 128.6, 135.0, 142.4, 159.1, 166.5, 178.6, 195.5 (C = O) ppm; Anal. Calcd for C_17_H_13_N_3_O_3_ (307.31): C, 66.44; H, 4.26; N, 13.67; Found: C, 66.67; H, 4.43; N, 13.94.

#### 2-Amino-5’-bromo-2’,5-dioxo-5,6,7,8-tetrahydrospiro[chromene-4,3’-indoline]-3-carbonitrile (6b)

IR (KBr): 3329 − 3143 (NH_2_, NH), 2187 (CN), 1720, 1674 (C = O) cm^− 1^; ^1^H NMR (400 MHz, DMSO-*d*_*6*_): δ 1.90–1.98 (m, 2 H, CH_2_), 2.23–2.27 (t, *J* = 6.4 Hz, 2 H, CH_2_), 2.64–2.67 (t, *J* = 6.0 Hz, 2 H, CH_2_), 6.74–6.76 (d, *J* = 8.2 Hz, 1H, ArH), 7.25–7.26 (d, *J* = 8.4 Hz,1H, ArH), 7.30–7.33 (m, 3 H, ArH + NH_2_, D_2_O exchangeable), 10.54 (s, 1H, NH, D_2_O exchangeable) ppm; ^13^C NMR (100 MHz, DMSO-*d*_*6*_): δ 20.1(C-7), 27.2 (C-8), 36.7 (C-6), 47.5 (C-4), 57.3 (C-3), 111.5, 111.7, 113.8, 117.7, 126.5, 131.3, 137.4, 141.8, 159.2, 167.0, 178.2, 195.7 (C = O) ppm; Anal. Calcd for C_17_H_12_BrN_3_O_3_ (386.21): C, 52.87; H, 3.13; N, 10.88; Found: C, 53.09; H, 3.40; N, 11.15.

#### 2-Amino-7,7-dimethyl-2’,5-dioxo-5,6,7,8-tetrahydrospiro[chromene-4,3’-indoline]-3-carbonitrile (6c)

IR (KBr): 3375 − 3313, 3143 (NH_2,_ NH), 2191 (CN), 1720 (C = O) cm^− 1^; ^1^H NMR (400 MHz, DMSO-*d*_*6*_): δ 1.00 (s, 6 H, two CH_3_), 2.08–2.20 (m, 2 H, CH_2_), 2.56–2.57 (m, 2 H, CH_2_), 6.78–6.80 (d, *J* = 7.6 Hz, 1H, ArH), 6.87–6.91 (t, *J* = 7.4 Hz, 1H, ArH), 6.97–6.99 (d, *J* = 7.1 Hz, 1H, ArH), 7.12–7.22 (m, 1H, ArH), 7.96 (s, 2 H, NH_2_, D_2_O exchangeable), 10.39 (s, 1H, NH, D_2_O exchangeable) ppm; ^13^C NMR (100 MHz, DMSO-*d*_*6*_): δ 27.4 (CH_3_), 28.0 (CH_3_), 31.2 (C-7), 32.4 (C-8), 36.2 (C-4), 47.2 (C-6), 58.0 (C-3), 109.7, 111.2, 117.8, 122.1, 123.4, 128.6, 134.8, 142.5, 159.2, 164.6, 178.5, 195.3 (C = O) ppm; Anal. Calcd for C_19_H_17_N_3_O_3_ (335.36): C, 68.05; H, 5.11; N, 12.53; Found: C, 67.91; H, 5.29; N, 12.74.

#### 2-Amino-5’-bromo-7,7-dimethyl-2’,5-dioxo-5,6,7,8-tetrahydrospiro[chromene-4,3’-indoline]-3-carbonitrile (6d)

IR (KBr): 3363 − 3290, 3158 (NH_2_, NH), 2194 (CN), 1728 (C = O) cm^− 1^,1681 (C = O) cm^− 1^; ^1^H NMR (400 MHz, DMSO-*d*_*6*_): δ 1.02 (s, 6 H, two CH_3_), 2.11–2.20 (m, 2 H, CH_2_), 2.49–2.62 (m, 2 H, CH_2_) 6.76–6.78 (d, *J* = 8.0 Hz, 1H, ArH), 7.21 (s, 1H, ArH), 7.31–7.33 (m, 3 H, ArH + NH_2_, D_2_O exchangeable, 10.55 (s, 1H, NH, D_2_O exchangeable) ppm; ^13^C NMR (100 MHz, DMSO-*d*_*6*_): δ 27.5 (CH_3_), 27.8 (CH_3_), 32.3 (C-7), 39.8 (C-8), 47.5 (C-4), 50.3 (C-6), 57.1 (C-3), 110.4, 111.8, 114.0, 117.6, 126.3, 131.5, 137.1, 141.6, 159.4, 165.4, 178.5, 196.0 (C = O) ppm; Anal. Calcd for C_19_H_16_BrN_3_O_3_ (414.26): C, 55.09; H, 3.89; N, 10.14; Found: C, 55.27; H, 4.05; N, 10.31.

#### Electrochemical synthesis of 2-amino-7,7-dimethyl-2’,5-dioxo-5,6,7,8-tetrahydrospiro[chromene-4,3’-indoline]-3-carbonitrile derivatives (6e-6 h)

A mixture of compound **7a**,** b** [38] (0.002 mmol) and cyclohexanedione **5a** or dimedone **5b** (0.002 mmol) was dissolved in ethanol (10 mL) in an undivided cell fitted with copper as a cathode and graphite as an anode. The reaction was conducted at 70 °C using a constant current of 30 mA. After completing the reaction as indicated by TLC, the reaction mixture was cooled, and water (20 mL) was added. The precipitate formed was filtered and recrystallized from ethanol.

#### Ethyl 2-amino-7,7-dimethyl-2’,5-dioxo-5,6,7,8-tetrahydrospiro[chromene-4,3’-indoline]-3-carboxylate (6e)

IR (KBr): 3371 − 3182 (NH_2_, NH), 1712, 1693, 1651 (C = O) cm^− 1^; ^1^H NMR (400 MHz, DMSO-*d*_*6*_): δ 0.78–0.82 (t, *J* = 7.1 Hz, 3 H, *CH*_*3*_CH_2_), 0.95 (s, 3 H, CH_3_), 1.02 (s, 3 H, CH_3_), 1.99–2.03 (d, *J* = 15.8 Hz, 1H), 2.13–2.17 (d, *J* = 15.8 Hz, 1H), 2.46–2.61 (m, 2 H, CH_2_), 3.66–3.75 (m, 2 H, CH_3_*CH*_*2*_), 6.66–6.68 (d, *J* = 7.5 Hz, 1H, ArH), 6.74–6.78 (t, *J* = 7.3 Hz, 1H, ArH), 6.82–6.84 (d, *J* = 6.9 Hz, 1H, ArH), 7.02–7.06 (t, *J* = 7.4 Hz, 1H, ArH), 7.85 (s, 2 H, NH_2_, D_2_O exchangeable), 10.13 (s, 1H, NH, D_2_O exchangeable ppm; ^13^C NMR (100 MHz, DMSO-*d*_*6*_): δ 13.5 (*CH*_*3*_CH_2_), 27.1(CH_3_), 28.2 (CH_3_), 32.0 (C-7), 39.9 (C-8), 47.0 (C-4), 51.1 (C-6), 59.3 (CH_3_*CH*_*2*_), 76.8 (C-3), 108.6, 113.5, 121.0, 122.6, 127.6, 136.4, 144.5, 159.5, 162.8, 168.1, 180.2, 195.1 (C = O) ppm; Anal. Calcd for C_21_H_22_N_2_O_5_ (382.42): C, 65.96; H, 5.80; N, 7.33; Found: C, 66.04; H, 5.97; N, 7.54.

#### Ethyl 2-amino-2’,5-dioxo-5,6,7,8-tetrahydrospiro[chromene-4,3’-indoline]-3-carboxylate (6f)

IR (KBr): 3360 − 3186 (NH_2_, NH), 1693, 1658, 1647 (C = O) cm^− 1^; ^1^H NMR (400 MHz, DMSO-*d*_*6*_): δ 0.78–0.81 (t, *J* = 7.0 Hz, 3 H, *CH*_*3*_CH_2_), 1.82–1.87 (m, 2 H, CH_2_), 2.10–2.24 (m, 2 H, CH_2_), 2.62–2.65 (t, *J* = 6.1 Hz, 2 H, CH_2_), 3.66–3.74 (m, 2 H, CH_3_*CH*_*2*_), 6.65–6.67 (d, *J* = 7.5 Hz, 1H, ArH), 6.74–6.77 (t, *J* = 7.4 Hz, 1H, ArH), 6.84–6.86 (d, 1H, *J* = 7.1 Hz, ArH), 7.02–7.06 (t, 1H, *J* = 7.5 Hz, ArH), 7.85 (s, 2 H, NH_2_, D_2_O exchangeable), 10.13 (s, 1H, NH, D_2_O exchangeable) ppm; ^13^C NMR (100 MHz, DMSO-*d*_*6*_): δ 13.5 (*CH*_*3*_CH_2_), 20.1 (C-7), 27.4 (C-8), 37.5 (C-6), 47.2 (C-4), 59.3 (CH_3_*CH*_*2*_), 76.8 (C-3), 108.5, 114.7, 120.9, 122.8, 127.5, 136.5, 144.4, 159.4, 164.6, 168.1, 180.3, 195.5 (C = O) ppm; Anal. Calcd for C_19_H_18_N_2_O_5_ (354.36): C, 64.40; H, 5.12; N, 7.91; Found: C, 64.67; H, 5.29; N, 8.13.

#### Ethyl 2-amino-5’-bromo-7,7-dimethyl-2’,5-dioxo-5,6,7,8-tetrahydrospiro[chromene-4,3’-indoline]-3-carboxylate (6 g)

IR (KBr): 3390 − 3278 (NH_2_, NH), 1727, 1685, 1651 (C = O) cm^− 1^; ^1^H NMR (400 MHz, DMSO-*d*_*6*_): δ 0.84–0.87 (t, 3 H, *J* = 7.2 Hz, *CH*_*3*_CH_2_), 0.96 (s, 3 H, CH_3_), 1.07 (s, 3 H, CH_3_), 2.05–2.15 (m, 2 H, CH_2_), 2.50–2.54 (m, 2 H, CH_2_), 3.71–3.75 (m, 2 H, CH_3_*CH*_*2*_), 6.64–6.66 (d, *J* = 8.4 Hz, 1H, ArH), 7.01 (s, 1H, ArH), 7.21–7.23 (d, *J* = 8.0 Hz, 1H, ArH), 7.93 (s, 2 H, NH_2_, D_2_O exchangeable), 10.33 (s, 1H, NH, D_2_O exchangeable) ppm; ^13^C NMR (100 MHz, DMSO-*d*_*6*_): δ 13.5 (*CH*_*3*_CH_2_), 27.5 (CH_3_), 27.8 (CH_3_), 32.0 (C-7), 47.3 (C-8), 51.0 (C-4), 56.5 (C-6), 59.4 (CH_3_*CH*_*2*_), 76.1 (C-3), 110.5, 112.5, 112.9, 125.5, 130.3, 138.9, 143.9, 159.6, 163.4, 167.9, 179.9, 195.3 (C = O) ppm; Anal. Calcd for C_21_H_21_BrN_2_O_5_ (461.31): C, 54.68; H, 4.59; N, 6.07; Found: C, 54.89; H, 4.67; N, 6.28.

#### Ethyl 2-amino-5’-bromo-2’,5-dioxo-5,6,7,8-tetrahydrospiro[chromene-4,3’-indoline]-3-carboxylate (6 h)

IR (KBr): 3336 − 3178 (NH_2_, NH), 1712, 1693, 1647 (C = O) cm^− 1^; ^1^H NMR (400 MHz, DMSO-*d*_*6*_): δ 0.80–0.84 (t, *J =* 7.2 Hz, 3 H, *CH*_*3*_CH_2_), 1.86–1.89 (t, *J* = 6.8 Hz, 2 H, CH_2_), 2.15–2.25 (m, 2 H, CH_2_), 2.62–2.65 (m, 2 H, CH_2_), 3.68–3.76 (m, 2 H, CH_3_*CH*_*2*_), 6.63–6.65 (d, *J* = 8.4 Hz, 1H, ArH), 7.04 (s, 1H, ArH), 7.21–7.23 (d, 1H, *J* = 8.4 Hz, ArH), 7.93 (s, 2 H, NH_2_, D_2_O exchangeable), 10.33 (s, 1H, NH, D_2_O exchangeable) ppm; ^13^C NMR (100 MHz, DMSO-*d*_*6*_): δ 13.5 (*CH*_*3*_CH_2_), 20.1 (C-7), 27.4 (C-8), 36.8 (C-6), 47.4 (C-4), 59.5 (CH_3_*CH*_*2*_), 76.2 (C-3), 110.4, 112.6, 113.9, 125.7, 130.3, 139.0, 143.9, 159.5, 165.3, 167.9, 180.1, 195.6 (C = O) ppm; Anal. Calcd for C_19_H_17_BrN_2_O_5_ (433.26): C, 52.67; H, 3.96; N, 6.47; Found: C, 52.89; H, 4.12; N, 6.71.

### Determination of acetylcholinesterase inhibition

Compounds **4b-4e**, **6a**, **6b**,** 6d**,** 6f**,** 6 h** were tested for their acetylcholinesterase inhibition according to the procedure of Elmann et al. [43] and Kia et al. [44]. The results are presented in Table 5.

### Preparation of samples

For the determination of percentage inhibition, standard donepezil HCl was prepared at a 0.5 µg/mL concentration and served as a positive control. Samples were dissolved initially in DMSO and then diluted in methanol to the final concentrations of 50 µg/mL and 500 µg/mL. For the determination of IC_50_, the standard donepezil was prepared at the following final concentrations: 0.0005, 0.005, 0.05, 0.5, and 5 µg/mL in water. Compounds **4e** and **6b** were dissolved initially in DMSO, then diluted in methanol at the following final concentrations: 31.25, 62.5, 125, 250, and 500 µg/mL. The enzyme acetylcholinesterase was purchased from Sigma-Aldrich from Electrophorus electricus. Cat number: 3389. The substrate acetylthiocholine iodide and the indicator 3,3′-dithiodipropionic acid di(*N*-hydroxy succinimide ester) (DTNB) were purchased from Sigma-Aldrich.

### Acetylcholinesterase inhibition assay

The assay followed the procedures outlined by Elmann et al. [43] and Kia et al. [44], with slight modifications. Briefly, 10µL of the indicator solution (0.4 mM in buffer (1): 100 mM tris buffer pH 7.5) was transferred to 96-well plate followed by 20µL of enzyme solution (acetylcholinesterase 0.02U/mL final concentration in buffer (2): 50 mM tris buffer pH 7.5 containing 0.1% bovine serum albumin). Then 20µL of the sample/standard solution was added, followed by 140µL of buffer (1). The mixture was allowed to stand for 15 min at room temperature. Afterwards, 10µL of the substrate (0.4 mM acetylcholine iodide buffer (1) was added immediately to all wells. The plate was kept in a dark chamber for 20 min at room temperature for incubation. At the end of the incubation period, the color was measured at 412 nm. The obtained data are represented as means ± SD.

### Determining the mode of Inhibition

To determine the mode of inhibition, the same assay used for assaying the inhibition of acetylcholinesterase was used with minor modification. The samples were incubated with the enzyme at the IC_50_ (0.51 and 0.84 mM for compounds **4e** and **6b**, respectively). Different concentrations of the substrate were added; 50 µM, 100 µM, 200 µM, 400 µM, 800 µM and 1600 µM concentrations were used. The absorbance was measured every 30 s for 20 min at 412 nm. Each concentration was assayed in triplicates.

### Microplate reader analysis

Data acquisition was performed with a FluoStar Omega microplate reader.

### Data analysis

The data was analyzed in Microsoft Excel^®^, and the IC_50_ values were determined using GraphPad Prism 6^®^ by applying a non-linear regression model (log(inhibitor) vs. normalized response–variable slope) to logarithmically transformed concentrations.

### Cytotoxicity study

Green monkey kidney (vero) was obtained from Nawah Scientific Inc., (Mokatam, Cairo, Egypt). Cells were maintained in DMEM media supplemented with 100 mg/mL of streptomycin, 100 units/mL of penicillin, and 10% of heat-inactivated fetal bovine serum in a humidified, 5% (v/v) CO_2_ atmosphere at 37 °C. Cell viability was assessed by the sulforhodamine B (SRB) assay [49, 50]. Aliquots of 100µL cell suspension (5 × 10^3^ cells) were in 96-well plates and incubated in complete media for 24 h. Cells were treated with another aliquot of 100 µL of media containing drugs at various concentrations. After drug exposure, cells were fixed by replacing media with 150 µL of 10% trichloroacetic acid (TCA) and incubated at 4 °C for 1 h. The TCA solution was removed, and the cells were washed 5 times with distilled water. Aliquots of 70 µL SRB solution (0.4% w/v) were added and incubated in a dark place at room temperature for 10 min. Plates were washed 3 times with 1% acetic acid and allowed to air-dry overnight. Then, 150 µL of 10 mM Tris base (pH ≈ 10.5) was added to dissolve protein-bound SRB stain; the absorbance was measured at 540 nm using an Infinite F50 microplate reader (TECAN, Switzerland).

### In *silico* prediction of physicochemical, ADME, and pharmacokinetic properties

The most potent synthesized derivatives, **4e** and **6b**, were analyzed using the SwissADME online platform (http://www.swissadme.ch) from the Swiss Institute of Bioinformatics (SIB) to obtain their physicochemical descriptors, ADME profiles, pharmacokinetic characteristics (log O/W, and activity against five CYP isoforms: CYP2C9, CYP1A2, CYP2D6, CYP3A4, and CYP2C19), and drug-like nature (evaluated by Lipinski, Ghose, Veber, Egan, and Muegge rules). The obtained data are presented in Table 6.

### Molecular docking study

**Fig. 11:**
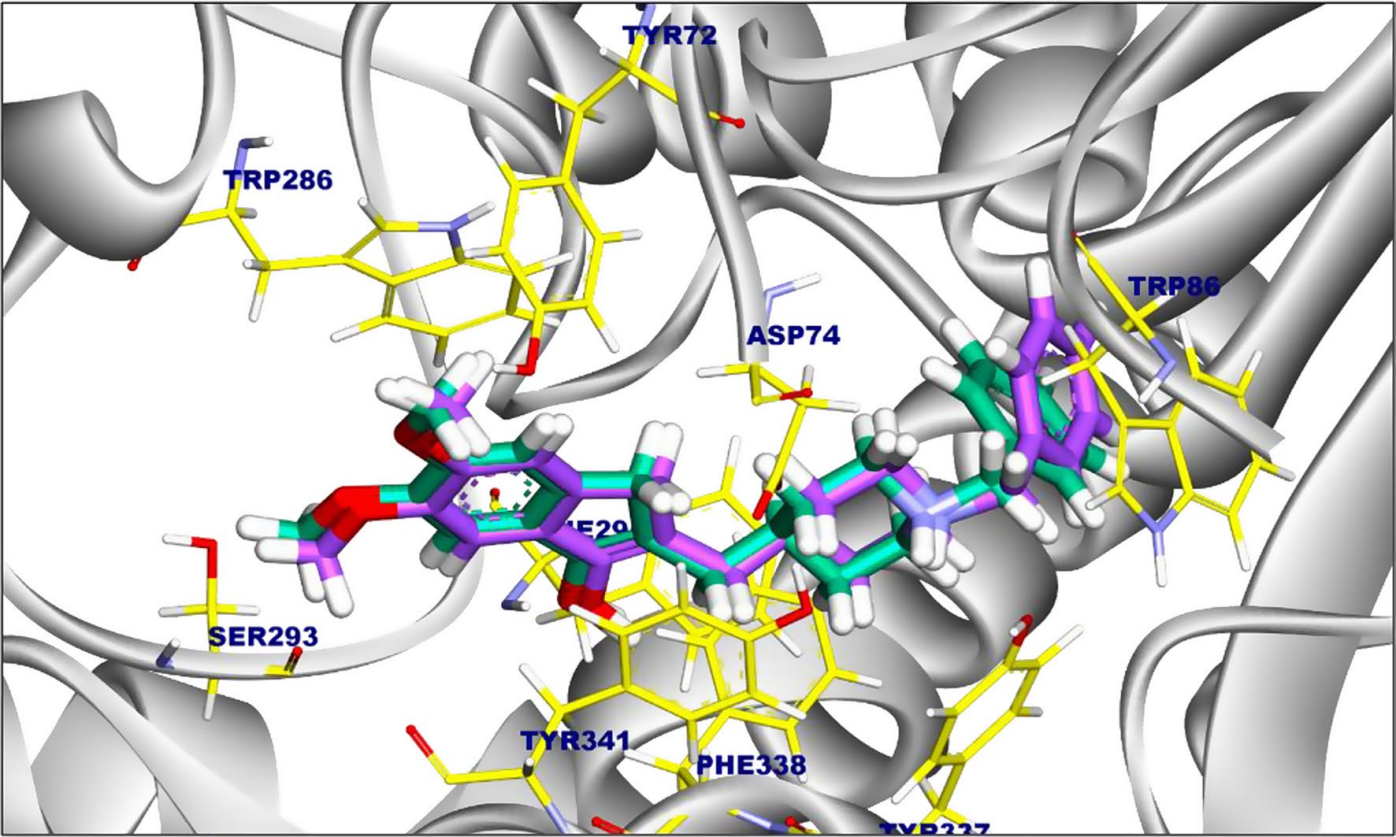
The 3D superimposition of donepezil inside human acetylcholinesterase with RMSD value of 0.65 Å, the original pose colored by purple and the redocked pose colored by green

The tested compounds were docked against human acetylcholinesterase (PDB code: 6O4W) [51] using Autodock Vina version 1.5.7. The co-crystallized ligand within the crystal protein (PDB code: 6O4W) obtained from the RCSB was used to generate the binding pocket. The protein complex was first cleaned of water molecules and other molecules that weren’t needed. After that, crystallographic disorders and unfilled valence atoms were corrected. The protein structure energy was reduced to minimum and saved as PDBQT file. Protonation and energy minimization were carried out and saved as PDBQT file. The docking process was conducted using Autodock Vina 1.5.7 software. To validate the docking approach employed in this investigation, donepezil (co-crystallized ligand) was self-docked inside human acetylcholinesterase (Fig. 11). Additionally, the docking scores of the best-fitted poses with the target protein were recorded, and 3D and 2D figures were generated using Discovery Studio 2024 visualizer [52].

## Conclusion

A simple, effective, and affordable technique for the synthesis of spiro[indoline-3,4′-pyrano[2,3-c]pyrazole] derivatives and 2-amino-7,7-dimethyl-2’,5-dioxo-5,6,7,8-tetrahydrospiro[chromene-4,3’-indoline]-3-carbonitrile derivatives was developed using a one-pot three component reaction in ethanol for 45 min at 70 °C using LiClO_4_ as an electrolyte and Cu/graphite as electrodes. The method afforded the target compounds in high yields using inexpensive and environmentally acceptable chemical reagents under non-hazardous reaction conditions. The compounds were tested for their acetylcholinesterase inhibition. Compounds **4e** and **6b** showed potent inhibition with IC_50_ values of 0.51 and 0.84 mM, respectively. The in silico study of the ADME properties of compounds **4e** and **6b** revealed a high bioavailability score. The most active compounds, **4e** and **6b** demonstrated minimal cytotoxicity in normal cell lines, with IC₅₀ values exceeding 100 µM and no significant morphological changes observed, highlighting their safety and selectivity. In addition, the molecular docking studies of compounds **4e** and **6b** supported the rationale of the study, showing that both compounds can serve as leads that can be optimized to enhance their biological activity and pharmacokinetic properties. A good correlation between the docking and kinetic studies affirmed that compounds **4e** and **6b** inhibited AChE competitively by binding at the CAS. The promising activity and the ease and economic synthesis of spirooxindole-pyranopyrazole and spirooxindole-chromene encourage further research in this pathway to obtain more potent and selective AChE inhibitors.

## Data Availability

The data supporting the conclusions of this study are available upon request from the authors.
